# How to Account for Past Selection When Maternal Effects Are Cascading

**DOI:** 10.1002/ece3.73725

**Published:** 2026-07-01

**Authors:** Rebecca B. Hoyle, Thomas H. G. Ezard, Bram Kuijper

**Affiliations:** ^1^ School of Mathematical Sciences University of Southampton Southampton UK; ^2^ School of Ocean and Earth Science University of Southampton Southampton UK; ^3^ Centre for Ecology and Conservation University of Exeter Exeter UK

**Keywords:** environmental change, evolutionary dynamics, maternal effects, maternal inheritance, phenotypic plasticity, quantitative genetics model

## Abstract

Many forms of maternal effects are said to be ‘cascading’, in which an individual's phenotype is partially a function of its mother's phenotype. The mother's phenotype is also partially a function of the grandmother's phenotype, and so each individual phenotype depends on the phenotypes of all its previous maternal lineage ancestors. In previous work we developed quantitative genetics models to assess the evolutionary consequences of such cascading maternal effects under pragmatic modelling assumptions. Here we show that the theoretical framework underlying our previous studies should be extended to treat past maternal states as being under selection in the current generation to account more consistently for the cascading nature of phenotypic parental effects. While accommodating selection of past maternal states does not significantly change the qualitative results of our previous studies, we find typically small quantitative changes in the evolving genetic components, offspring phenotype and fitness. The extended framework offers a conceptually more consistent approach that can inform future quantitative genetics models of parental effects. In particular this new formulation captures the impact of genetic covariances between current and past states on the evolutionary dynamics, and provides a flexible framework that can be adapted to analyse transgenerational influences from any combination of ancestors.

## Graphical abstract

Many forms of maternal effects are said to be ‘cascading’, in which an individual's phenotype is partially a function of its mother's phenotype and hence also partially a function of the phenotypes of its previous ancestors. We develop a quantitative genetics modelling framework that treats past maternal states as being under selection in the current generation to account more consistently for the cascading nature of phenotypic parental effects. This extended framework can be adapted to analyse transgenerational influences from any combination of ancestors.

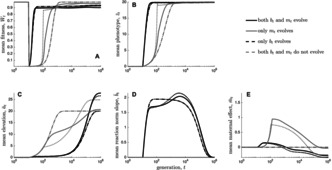

## 1 Introduction

Maternal effects, where the maternal phenotype or genotype exerts a causal influence on the offspring phenotype other than by the direct transmission of genes (Mousseau and Fox 1998; Wolf and Wade 2009; Van Dooren et al. 2026), are common, acting on diverse traits and organisms such as the age at maturity in springtails (Janssen et al. 1988), clutch size in collared flycatchers (Schluter and Gustafsson 1993) and juvenile growth in red squirrels (McAdam and Boutin 2004). Analogous effects arising from the influences of other ancestors are also possible, for example paternal effects (e.g., Crean and Bonduriansky (2014), Yang and Rando (2025)) and grandmaternal effects (e.g., Hafer et al. (2011)).

Maternal effects arising from the maternal phenotype have the potential to cascade down multiple generations. For example, a nongenomic pathway has been demonstrated for the transmission of high licking and grooming behaviour in rats (Francis et al. (1999); see also Cameron et al. (2008) and Cameron (2011)): the daughters of high licking and grooming mothers themselves lick and groom their own offspring more than the daughters of low licking and grooming mothers do. Thus a tendency to high licking and grooming can be passed down the generations from mothers to daughters, granddaughters and beyond, in addition to any direct genetic influence of the inherited genes on licking and grooming behaviour. This is an example of a positive maternal effect, where, all other contributions to the offspring phenotype being equal, the offspring phenotype is larger for larger maternal phenotype. Positive cascading maternal effects can accelerate the response to selection, leading to faster adaptation than under direct genetic transmission alone, at the cost of greater phenotypic variance and lower equilibrium population mean fitness. Conversely negative cascading maternal effects, where the offspring phenotype is smaller for larger maternal phenotype, can slow the response to selection, and if they are not too strong can also reduce phenotypic variance and increase population mean fitness at equilibrium (Hoyle and Ezard 2012).

The evolution of cascading maternal effects, where an individual's phenotype is directly influenced by its mother's phenotype and hence indirectly by the phenotypes of its previous maternal lineage ancestors, is especially interesting (Wolf et al. 1998; Wolf and Wade 2009). Not only do the maternal effects themselves transmit information from one generation to another outside of strict genetic inheritance, but the conditions under which they evolve and the form that they take shed light on the circumstances in which such additional inheritance mechanisms confer evolutionary advantage. These questions have been the focus of a series of models, notably seminal work by Kirkpatrick and Lande (1989) based in the theoretical framework of Lande (1979).

In a series of earlier articles (Hoyle and Ezard 2012; Ezard et al. 2014; Prizak et al. 2014; Kuijper and Hoyle 2015), we built upon these models to consider how cascading maternal effects and within‐generation phenotypic plasticity combine to deliver an evolving phenotype in response to environmental change, using a similar approach to the Lande (2009) model of the Baldwin effect. We introduced a theoretical framework based in quantitative genetics to describe the evolution of the phenotype

(1) $$z_{t}=a_{t}+b_{t}\varepsilon_{t-\tau }+m_{t}z_{t-1}^{*}+e_{t},$$

for a single trait that affects itself maternally, where t labels the generation, $z_{t}$ is the individual adult phenotype, $a_{t}$ is the elevation in the reference environment ε=0, $b_{t}$ is the slope of the reaction norm of within‐generation plasticity, $\varepsilon_{t}$ is the common environment at time t, τ is the lag between a critical period in which juvenile development is affected by the environment and the time when selection acts on the adult, $m_{t}$ is the strength of the maternal effect, $z_{t-1}^{*}$ is the maternal phenotype after selection in generation t−1, and $e_{t}∼N\left(0,\sigma_{e}\right)$ is the independent residual component of phenotypic variation. The genetic components $a_{t}$, $b_{t}$ and $m_{t}$ are assumed to be normally distributed and independent. Generations are assumed to be discrete and non‐overlapping, and reproduction is assumed to be sexual with mating at random.

In our original framework we considered only the three genetic components $a_{t}$, $b_{t}$ and $m_{t}$ in the current generation to be evolving, treating the history of past states as fixed. Following the Lande framework (Lande 1979, 2009) we previously set

$$\left(\begin{array}{c} \Delta {\bar{a}}_{t}\\ \Delta {\bar{b}}_{t}\\ \Delta {\bar{m}}_{t} \end{array}\right)=G\nabla \ln\bar{W},$$

where the overbars denote the population mean, W is fitness, G is the genetic variance–covariance matrix and the genetic values a, b and m are mutually independent so that

$$G=\left(\begin{array}{ccc} G_{\mathrm{aa}} & 0 & 0\\ 0 & G_{\mathrm{bb}} & 0\\ 0 & 0 & G_{\mathrm{mm}} \end{array}\right).$$

However, a fresh consideration of our models has led us to realise that when maternal effects are cascading we should treat the history of past states as also evolving. This may seem counterintuitive, because the past cannot be changed in the present. However, in the presence of maternal effects each contemporary individual carries influences from past maternal, grand‐maternal and earlier maternal lineage phenotypes. When selection acts on the current phenotype in the current generation, it changes the distribution of these ‘carried’ past states in the current population. Thus selection in the current generation also selects the past lineages present in the current population. The genetic values of the line of mothers, grandmothers and great‐grandmothers belonging to the members of the current generation who are selected for become enriched in the distribution of past states, while the genetic values of those whose descendants are selected against drop out of the history of past states. These changes in the distribution of past states contribute to changes in the population mean fitness and affect the form of the evolution equations for the genetic components. Incorporating the effects of evolving past states updates our original framework in three main ways. First, the impact of the maternal generation on the speed of evolution of the current generation genetic components is reduced by a factor of two, because the maternal genetic component is now treated as a covarying ‘carried’ past state under selection with covariance equal to a half the current generation genetic variance (see Section 2). Second, the speed of evolution of the current generation genetic components depends explicitly on contributions from the whole history of earlier generations, rather than only implicitly through the values of the current generation genetic components as is the case when ‘carried’ maternal lineage genetic components are not treated as covarying. Third and finally, the change in the current phenotype at selection includes contributions from updates to the means of past maternal phenotypes, and additionally from updates to the residual component of phenotypic variation which we also include in our extended framework.

Here we compare updated results in our new extended framework with our original findings. In short, we find some typically small quantitative changes, but qualitatively the models behave very similarly. While we have not repeated every analysis and there are differences of detail, our major findings and conclusions appear broadly unchanged. We highlight some interesting conceptual differences between the two approaches which may be informative for future work.

Our extended framework is flexible, allowing for more complex patterns of maternal lineage phenotypic influence on the offspring phenotype: for example in Section 4 we model the case where both the maternal and grandmaternal phenotypes directly contribute to the offspring phenotype. It could also be extended further to incorporate paternal and mixed lineage effects, thus allowing for bespoke parental effects models that incorporate the direct influence of any ancestors of interest on the offspring phenotype.

In the next section we introduce our extended theoretical framework before moving on to examine its consequences for the cases of fixed maternal effects, fixed maternal and grandmaternal effects, and evolving maternal effects.

## 2 Extended Theoretical Framework

We consider a situation where fitness depends not only on the genetic components $a_{t}$, $b_{t}$ and $m_{t}$ in the current generation, but also on the history of maternal phenotypes $z_{t-n}^{*\cdots *}$, and hence the history of maternal lineage genetic components $a_{t-n}^{*\cdots *}$, $b_{t-n}^{*\cdots *}$ and $m_{t-n}^{*\cdots *}$ after selection, where t is the current generation and n=1,2,… counts the past generations. We use this completely general formulation to provide a flexible framework, but we can also see by expanding $z_{t-1}^{*}$ that even the simplest case of cascading maternal effects represented by Equation (1) involves the full history of maternal phenotypes:
(2)

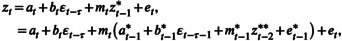

where $z_{t-2}^{**}$ and successively older phenotypes can be expanded in turn.

Following Lande (1979), whose workflow we adapt here, the distribution of genetic components is given by

$$g\left(x\right)=\sqrt{{\left(2\pi \right)}^{-3(N+1)}|G^{-1}|}\exp\left\{-\frac{1}{2}{\left(x-\bar{x}\right)}^{T}G^{-1}\left(x-\bar{x}\right)\right\},$$

where in our case

$$x=\left(\begin{array}{c} a_{t}\\ b_{t}\\ m_{t}\\ a_{t-1}^{*}\\ b_{t-1}^{*}\\ m_{t-1}^{*}\\ a_{t-2}^{**}\\ b_{t-2}^{**}\\ m_{t-2}^{**}\\ \vdots \end{array}\right),$$

and N is the number of past generations retained in the formalism, which in practice will be finite, though for simplicity we initially write the equations without an explicit final past generation and take the limit as N→∞ in the final update equations. Here the two asterisks on $a_{t-2}^{**}$, $b_{t-2}^{**}$ and $m_{t-2}^{**}$ indicate that the grandmaternal genetic components have passed through two rounds of selection: one in the grandmaternal generation and one in the maternal generation. Similarly the superscripts on $a_{t-n}^{*\cdots *}$ $b_{t-n}^{*\cdots *}$ and $m_{t-n}^{*\cdots *}$ indicate that they carry n asterisks to show that they have been passed through n rounds of selection. We will make use of this additional precision in our notation later when considering grandmaternal states.

Under weak selection we have

$$G\approx \left(\begin{array}{ccccccc} G_{\mathrm{aa}} & 0 & 0 & \frac{1}{2}G_{\mathrm{aa}} & 0 & 0 & \ldots \\ 0 & G_{\mathrm{bb}} & 0 & 0 & \frac{1}{2}G_{\mathrm{bb}} & 0 & \ldots \\ 0 & 0 & G_{\mathrm{mm}} & 0 & 0 & \frac{1}{2}G_{\mathrm{mm}} & \ldots \\ \frac{1}{2}G_{\mathrm{aa}} & 0 & 0 & G_{\mathrm{aa}} & 0 & 0 & \ldots \\ 0 & \frac{1}{2}G_{\mathrm{bb}} & 0 & 0 & G_{\mathrm{bb}} & 0 & \ldots \\ 0 & 0 & \frac{1}{2}G_{\mathrm{mm}} & 0 & 0 & G_{\mathrm{mm}} & \ldots \\ \vdots & \vdots & \vdots & \vdots & \vdots & \vdots & \ddots \end{array}\right),$$

where we have used the weak selection (or near equilibrium) approximations $\mathrm{Cov}\left(a_{t},a_{t-1}^{*}\right)\approx \frac{1}{2}G_{\mathrm{aa}}$, $\mathrm{Var}\left(a_{t-1}^{*}\right)\approx G_{\mathrm{aa}}$, $\mathrm{Cov}\left(b_{t},b_{t-1}^{*}\right)\approx \frac{1}{2}G_{\mathrm{bb}}$, $\mathrm{Var}\left(b_{t-1}^{*}\right)\approx G_{\mathrm{bb}}$, $\mathrm{Cov}\left(m_{t},m_{t-1}^{*}\right)\approx \frac{1}{2}G_{\mathrm{mm}}$, $\mathrm{Var}\left(m_{t-1}^{*}\right)\approx G_{\mathrm{mm}}$. In the continuation of the matrix represented by dots, we have $\mathrm{Cov}\left(a_{t},a_{t-n}^{*\cdots *}\right)\approx \frac{1}{2^{n}}G_{\mathrm{aa}}$, $\mathrm{Cov}\left(a_{t-m}^{*\cdots *},a_{t-n}^{*\cdots *}\right)\approx \frac{1}{2^{|n-m|}}G_{\mathrm{aa}}$, $\mathrm{Var}\left(a_{t-n}^{*\cdots *}\right)\approx G_{\mathrm{aa}}$ and the equivalent for b and m. We approximate the remaining covariances by $\mathrm{Cov}\left(*,*\right)\approx 0$. We assume that G is constant, and neglect any contributions to G from the impact of past selection. The factors of powers of one half arise because each individual has two parents and under weak selection the covariance between the individual's genetic component and that of one of its parents is approximately one half the current generation genetic variance. The G matrix ensures the correct weighting of the influence of each ancestor in the analysis that follows.

Then we calculate the population mean fitness according to

$$\bar{W}=\int g\left(x\right)\tilde{W}\left(x\right)dx,$$

where $\tilde{W}\left(x\right)$ is the mean fitness of individuals with genetic component history x.

Note that we also have

(3) $$\bar{W}=\int \int g\left(x\right)f\left(e\right)W^{†}\left(x,e\right)dxde,$$

where

$$f\left(e\right)=\sqrt{{\left(2\pi \right)}^{-\left(N+1\right)}|E^{-1}|}\exp\left\{-\frac{1}{2}{\left(e-\bar{e}\right)}^{T}E^{-1}\left(e-\bar{e}\right)\right\}$$

is the (normal) distribution of the vector $e=\left(e_{t},e_{t-1}^{*},e_{t-2}^{**},\ldots \right)$ of independent residual components of phenotypic variation in each generation (with asterisks once again indicating the number of rounds of selection through which their influence has passed), and where $W^{†}\left(x,e\right)$ is the fitness of an individual with genetic component history x and residual history e. Similarly to Lande (1979), we assume that the $e_{t}∼N\left(0,\sigma_{e}\right)$ for all generations t, and are independent of each other (i.e., across generations) and of the genetic component history x. Note that the means of past residuals $e_{t-n}^{*\cdots *}$ are not constrained to be zero after selection, however we make the weak selection or near equilibrium approximations that their variances are approximately $\sigma_{e}^{2}$, and they are independent of each other, of $e_{t}$ and of x, so that the distributions of x and e are independent as written in Equation (3), and E is diagonal. We assume that E is constant, and neglect any contributions to E or any covariances between the elements of x and e arising from the impact of past selection. Thus we have

$$\tilde{W}\left(x\right)=\int f\left(e\right)W^{†}\left(x,e\right)de.$$

We do not include here explicitly the dependence of the fitness on the environment $\varepsilon_{t}$ as we assume that the environment does not vary across the population.

For later use we note that the population mean fitness can also be taken over the distribution of phenotypes:

$$\bar{W}=\int p\left(z\right)W\left(z\right)dz,$$

where $p\left(z\right)$ is the phenotype distribution, and $W\left(z\right)$ is the fitness of phenotype z.

Now

$$\begin{array}{c} \nabla \ln\bar{W}=\frac{1}{\bar{W}}\nabla \bar{W},\\ \;=\frac{1}{\bar{W}}G^{-1}\int \left(x-\bar{x}\right)g\left(x\right)\tilde{W}\left(x\right)dx\\ \;=G^{-1}\frac{1}{\bar{W}}\int xg\left(x\right)\tilde{W}\left(x\right)dx-G^{-1}\bar{x}\\ \;=G^{-1}\Delta \bar{x}. \end{array}$$

Thus we have

(4) $$\left(\begin{array}{c} \Delta {\bar{a}}_{t}\\ \Delta {\bar{b}}_{t}\\ \Delta {\bar{m}}_{t}\\ \vdots \end{array}\right)=G\nabla \ln\bar{W}$$

(5)

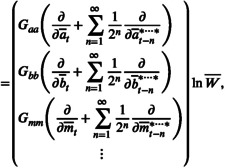

which is equivalent to eq. (7b) of Lande (1979). This allows us to calculate the mean genetic values in the offspring generation according to

$$\begin{array}{c} {\bar{a}}_{t+1}\;={\bar{a}}_{t}^{*}={\bar{a}}_{t}+\Delta {\bar{a}}_{t},\\ {\bar{b}}_{t+1}\;={\bar{b}}_{t}^{*}={\bar{b}}_{t}+\Delta {\bar{b}}_{t},\\ {\bar{m}}_{t+1}={\bar{m}}_{t}^{*}={\bar{m}}_{t}+\Delta {\bar{m}}_{t}, \end{array}$$

where the means in the terms ${\bar{a}}_{t+1}$ and ${\bar{a}}_{t}^{*}$ are taken over the offspring generation t+1 and the means in the terms ${\bar{a}}_{t}$ and $\Delta {\bar{a}}_{t}$ are taken over the current generation t, and the equivalent also holds for b and m; see Appendix A.

From Equation (2) we see that in order to calculate the mean value of the phenotype after selection in the current generation, ${\bar{z}}_{t}^{*}$, we need to know how the distributions of the past states and the residuals change at selection. For example, considering the case of evolutionarily constant maternal effects $m_{t}$ $\left(\equiv m\right)$ for simplicity, we have

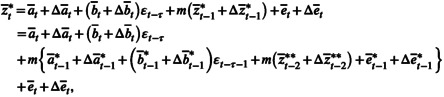

and ${\bar{z}}_{t-2}^{**}$ can be expanded further in terms of the past states of earlier ancestors. Thus we need expressions for $\Delta {\bar{a}}_{t-n}^{*\ldots *}$, $\Delta {\bar{b}}_{t-n}^{*\cdots *}$, $\Delta {\bar{m}}_{t-n}^{*\cdots *}$, $\Delta {\bar{e}}_{t}$ and $\Delta {\bar{e}}_{t-n}^{*\cdots *}$. Note that the mean in ${\bar{z}}_{t}^{*}$ is taken over the population in generation t+1, as selection is the point at which the next generation becomes “current” in our framework and population means are taken over the current generation.

The remaining rows of $G\nabla \ln\bar{W}$ in Equation (5) give the updates to the population mean of past states at selection in the current generation, owing to the change in distribution of the past states:

(6) $$\Delta {\bar{a}}_{t-n}^{*\cdots *}=\frac{1}{2^{n}}G_{\mathrm{aa}}\frac{\partial \ln\bar{W}}{\partial {\bar{a}}_{t}}+G_{\mathrm{aa}}\sum_{k=1}^{\infty }\frac{1}{2^{|n-k|}}\frac{\partial \ln\bar{W}}{\partial {\bar{a}}_{t-k}^{*\cdots *}},$$

(7) $$\Delta {\bar{b}}_{t-n}^{*\cdots *}=\frac{1}{2^{n}}G_{\mathrm{bb}}\frac{\partial \ln\bar{W}}{\partial {\bar{b}}_{t}}+G_{\mathrm{bb}}\sum_{k=1}^{\infty }\frac{1}{2^{|n-k|}}\frac{\partial \ln\bar{W}}{\partial {\bar{b}}_{t-k}^{*\cdots *}},$$

(8) $$\Delta {\bar{m}}_{t-n}^{*\cdots *}=\frac{1}{2^{n}}G_{\mathrm{mm}}\frac{\partial \ln\bar{W}}{\partial {\bar{m}}_{t}}+G_{\mathrm{mm}}\sum_{k=1}^{\infty }\frac{1}{2^{|n-k|}}\frac{\partial \ln\bar{W}}{\partial {\bar{m}}_{t-k}^{*\cdots *}}.$$

Equations ((6), (7), (8)) reflect evolutionary change in the components of the current generation's phenotype that were contributed by an ancestor n generations ago. For example, Equation (6) reflects contemporary change (between generations t and t+1) of (the mean over the current population of) the elevation $a_{t-n}^{*\cdots *}$ expressed by a maternal lineage ancestor n generations ago that is now part of an individual's phenotype. The first term on the right‐hand side reflects how contemporary selection (in generation t) on the elevation expressed by the current generation, $a_{t}$, affects change in the current generation's phenotypic component of $a_{t-n}^{*\cdots *}$. Selection on $a_{t}$ affects the distribution of $a_{t-n}^{*\cdots *}$ as $a_{t}$ and $a_{t-n}^{*\cdots *}$ are genetically correlated, as measured by the covariance $\frac{1}{2^{n}}G_{\mathrm{aa}}$, which reflects that $a_{t}$ is genetically descendant from $a_{t-n}^{*\cdots *}$. The second part on the right‐hand side has a similar interpretation, and reflects how contemporary selection on the current generation's phenotypic component of the elevation $a_{t-k}^{*\cdots *}$ contributed by ancestors k generations ago affects evolutionary change in the current generation's phenotypic component of $a_{t-n}^{*\cdots *}$, again because $a_{t-k}^{*\cdots *}$ and $a_{t-n}^{*\cdots *}$ are statistically associated through common descent, weighted by the genetic correlation $\frac{1}{2^{|n-k|}}G_{\mathrm{aa}}$.

Note that, by definition, updates to the maternal effect coefficients ${\bar{m}}_{t}$ and ${\bar{m}}_{t-n}^{*\cdots *}$ at selection in the current generation update the weighting of past states in the current generation phenotype after selection and in the phenotype of the next generation.

The principal differences from our previous models arise from the factor of $\frac{1}{2}$ in front of the derivatives with respect to the maternal genetic components owing to the genetic correlations described above, and from the influence of the derivatives with respect to genetic components in earlier generations, both in Equation (5). Previously in earlier work (Hoyle and Ezard 2012; Ezard et al. 2014; Prizak et al. 2014; Kuijper and Hoyle 2015) that can be considered as simplified heuristic models neglecting the evolution of past states, we effectively substituted ${\bar{a}}_{t-1}^{*}={\bar{a}}_{t}$, ${\bar{b}}_{t-1}^{*}={\bar{b}}_{t}$ and ${\bar{m}}_{t-1}^{*}={\bar{m}}_{t}$ into $\ln\bar{W}$ and differentiated with respect to the genetic components in the present generation only. However this leads to the loss of the factor of $\frac{1}{2}$ in the influence of the maternal generation and loss of the influence of previous generations on the speed of evolution. The heuristic aspect of these models was the (effective) substitution of ${\bar{a}}_{t-1}^{*}={\bar{a}}_{t}$, ${\bar{b}}_{t-1}^{*}={\bar{b}}_{t}$ and ${\bar{m}}_{t-1}^{*}={\bar{m}}_{t}$ into $\ln\bar{W}$. Without it the models would simply have neglected the impact of evolving past states entirely; including the substitution captured the influence of the maternal generation on the speed of evolution, but overstated it.

In order to calculate the change to the current phenotype at selection correctly, we also have to take into account the impact of selection on the residual components of phenotypic variation, e. Analogously to the calculation above for the genetic component vector x, we have for e,

$$\nabla_{e}\ln\bar{W}=E^{-1}\Delta \bar{e},$$

where $E=\sigma_{e}^{2}I$. Thus we have

(9) $$\left(\begin{array}{c} \Delta {\bar{e}}_{t}\\ \Delta {\bar{e}}_{t-1}^{*}\\ \vdots \end{array}\right)=\left(\begin{array}{c} \sigma_{e}^{2}\frac{\partial }{\partial {\bar{e}}_{t}}\\ \sigma_{e}^{2}\frac{\partial }{\partial {\bar{e}}_{t-1}^{*}}\\ \vdots \end{array}\right)\ln\bar{W}.$$

As we will see later, including the update to e gives

$${\bar{z}}_{t}^{*}=\bar{z_{t}}+\sigma_{z}^{2}\frac{d\left(\ln\bar{W}\right)}{d{\bar{z}}_{t}},$$

where ${\bar{z}}_{t}^{*}$ is the phenotype after selection in the current generation and $\sigma_{z}^{2}$ is the phenotypic variance. In the notation of Kirkpatrick and Lande (1989), this can be written

$${\bar{z}}_{t}^{*}=\bar{z_{t}}+\mathrm{P\beta},$$

where $P=\sigma_{z}^{2}$ and where

$$\beta =\frac{d\left(\ln\bar{W}\right)}{d{\bar{z}}_{t}}$$

is the selection gradient. Thus considering e also to be evolving, in the sense of its mean changing at selection, is important to fully align the formalism used here and in Lande (2009) with that of Lande (1979) and Kirkpatrick and Lande (1989) (even in the case of no maternal effects).

In Appendix A we explore some additional subtleties of the updates to past states, notably that we assume the distribution of the genetic component history, x, and the mean fitness of a given genetic component history to be the same in males and females, and highlight that our models hold true for both viability and fertility selection and so are more general than we had previously claimed.

The framework could be extended further to incorporate paternal effects alongside maternal effects by introducing new past state variables related to the paternal line, labelled with † rather than *, with cross‐lineage past states being labelled with the correct sequence of * and † to indicate the ancestral line to each past state and so keep track of relatedness. Covariance matrices would then be adapted to incorporate all the relevant relatedness information for the family tree, and all relevant intermediate past states would be included in the formalism. If only effects of the paternal line are required then the formalism used here for maternal effects can be adopted without alteration, but with * now denoting a past paternal state.

## 3 Fixed Maternal Effects

In this section we will explore the impact of the extended framework when the maternal effect $m_{t}$ is considered fixed, in other words evolutionarily constant, as in Hoyle and Ezard (2012) and Ezard et al. (2014).

When $m_{t}=m$ is fixed the remaining genetic components ${\bar{a}}_{t}$ and ${\bar{b}}_{t}$ update according to

$$\left(\begin{array}{c} \Delta {\bar{a}}_{t}\\ \Delta {\bar{b}}_{t} \end{array}\right)=\left(\begin{array}{c} G_{\mathrm{aa}}\left(\frac{\partial }{\partial {\bar{a}}_{t}}+\sum_{n=1}^{\infty }\frac{1}{2^{n}}\frac{\partial }{\partial {\bar{a}}_{t-n}^{*\cdots *}}\right)\\ G_{\mathrm{bb}}\left(\frac{\partial }{\partial {\bar{b}}_{t}}+\sum_{n=1}^{\infty }\frac{1}{2^{n}}\frac{\partial }{\partial {\bar{b}}_{t-n}^{*\cdots *}}\right) \end{array}\right)\ln\bar{W}.$$

Following Hoyle and Ezard (2012) the fitness is given by

$$W\left(z_{t}\right)=W_{\max}\exp\left\{-\frac{{\left(z_{t}-\theta_{t}\right)}^{2}}{2\omega^{2}}\right\},$$

where $\theta =A+B\varepsilon_{t}$ is the optimum phenotype and A, B, $W_{\max}$ and ω are constants. Thus the population mean fitness is given by

$$\bar{W}\left({\bar{z}}_{t}\right)=\int p\left(z_{t}\right)W\left(\varepsilon_{t},z_{t}\right)dz_{t}=W_{\max}\sqrt{\gamma \omega^{2}}\exp\left\{-\frac{\gamma }{2}{\left({\bar{z}}_{t}-\theta_{t}\right)}^{2}\right\},$$

where $z_{t}$ is assumed to be normally distributed with variance $\sigma_{z_{t}}^{2}$ and $\gamma =1/\left(\omega^{2}+\sigma_{z_{t}}^{2}\right)$, where for weak selection γ is assumed to be small. The population mean phenotype is given by

$${\bar{z}}_{t}={\bar{a}}_{t}+{\bar{b}}_{t}\varepsilon_{t-\tau }+m{\bar{z}}_{t-1}^{*}+{\bar{e}}_{t},$$

and so we have

$$\begin{array}{c} \Delta {\bar{a}}_{t}=-\gamma G_{\mathrm{aa}}\left({\bar{a}}_{t}-A+{\bar{b}}_{t}\varepsilon_{t-\tau }-B\varepsilon_{t}+m{\bar{z}}_{t-1}^{*}\right)\sum_{n=0}^{\infty }{\left(\frac{m}{2}\right)}^{n},\\ \Delta {\bar{b}}_{t}=-\gamma G_{\mathrm{bb}}\left({\bar{a}}_{t}-A+{\bar{b}}_{t}\varepsilon_{t-\tau }-B\varepsilon_{t}+m{\bar{z}}_{t-1}^{*}\right)\sum_{n=0}^{\infty }{\left(\frac{m}{2}\right)}^{n}\varepsilon_{t-\tau -n}, \end{array}$$

where we have substituted ${\bar{e}}_{t}=0$ because the mean residual is zero. The sum in the equation for $\Delta {\bar{a}}_{t}$ can be evaluated exactly

$$\sum_{n=0}^{\infty }{\left(\frac{m}{2}\right)}^{n}=\frac{1}{1-\frac{m}{2}}=\frac{2}{2-m},$$

but the sum in the equation for $\Delta {\bar{b}}_{t}$ cannot be, owing to the terms $\varepsilon_{t-\tau -n}$, and has to be truncated by only including terms up to a certain power of m (corresponding to a specific number of generations). For ∣m∣<1 (which is the range of m around zero such that the phenotypic variance does not diverge—see Hoyle and Ezard (2012)) truncation of the sum should provide an acceptable approximation. Note that if the whole history of past environments $\varepsilon_{t-\tau -n}$ is known, then in principle the sum can be evaluated without truncation. Similarly if the environment is constant so that $\varepsilon_{t-\tau -n}\equiv \varepsilon$, where ε is a constant, then the sum can be evaluated as in the $\Delta {\bar{a}}_{t}$ equation. However, to make analytical progress in the case of a general changing environment truncation of the sum is a pragmatic approach.

For the most direct comparison with our original results, where the influences of only the current and parental generations were included in the corresponding factors, we truncate both sums at $O\left(m\right)$ giving

(10) $$\Delta {\bar{a}}_{t}=-\gamma G_{\mathrm{aa}}\left({\bar{a}}_{t}-A+{\bar{b}}_{t}\varepsilon_{t-\tau }-B\varepsilon_{t}+m{\bar{z}}_{t-1}^{*}\right)\left(1+\frac{m}{2}\right),$$

(11) $$\Delta {\bar{b}}_{t}=-\gamma G_{\mathrm{bb}}\left({\bar{a}}_{t}-A+{\bar{b}}_{t}\varepsilon_{t-\tau }-B\varepsilon_{t}+m{\bar{z}}_{t-1}^{*}\right)\left(\varepsilon_{t-\tau }+\frac{m}{2}\varepsilon_{t-\tau -1}\right).$$

Truncation of the sums at $O\left(m\right)$, that is, neglecting terms in $m^{k}$ where k>1, is equivalent to neglecting the influence of generations prior to the maternal generation on the multiplicative factor that governs the speed of evolution. It is possible to include the influence of previous generations if desired by taking more terms in the sum before truncation. Note that while we truncate the sums, we nonetheless retain in the overall update equations $O\left(m^{2}\right)$ terms arising from the product of the term $m{\bar{z}}_{t-1}^{*}$ in the first bracket and the term in m in the second bracket; this preserves the common structure of the equations and so helps with analysis and interpretation of the model. We shall adopt this practice throughout. Similarly we retain the dependence of γ on m that arises from the phenotypic variance.

To complete the updates at each generation we also need to know how the phenotype itself updates at selection in generation t. There is a subtlety here that is revealed in our extended theoretical framework: where we update ${\bar{z}}_{t}$ after selection in the current generation to get ${\bar{z}}_{t}^{*}$ we need to include changes in ${\bar{z}}_{t-1}^{*}$ that arise at selection in the current generation owing to changes in the distributions of ‘carried’ past states and residuals, and we also need to include the update to ${\bar{e}}_{t}$ at selection. To first order in m we find

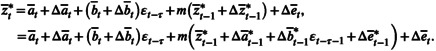




From Equations (6), (7) and (9) above we have:

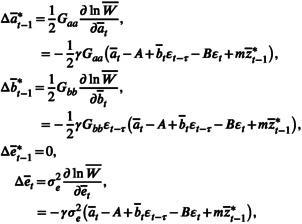

to $O\left(1\right)$, where we have substituted ${\bar{e}}_{t}=0$, and have retained the $m{\bar{z}}_{t-1}^{*}$ terms in the brackets to preserve the common structure of the equations. Note that while $\Delta {\bar{e}}_{t-1}^{*}$ is not identically zero, it has no $O\left(1\right)$ terms and so is approximated to zero here. Thus to first order in m we have 
(12)

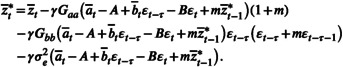




Here we can see a connection with our original way of calculating the updates: at selection in generation t, changes in the vector of genetic components contribute the same amount to the update from ${\bar{z}}_{t}$ to ${\bar{z}}_{t}^{*}$ as was the case using our previous formulation (Hoyle and Ezard (2012) eq. 2.10–2.12) where all of the update came from the terms $\Delta {\bar{a}}_{t}$ and $\Delta {\bar{b}}_{t}$. However, because our earlier model attributed the contribution from updating the past states to updates in the current states ${\bar{a}}_{t}$ and ${\bar{b}}_{t}$, the dynamics for the current states and for ${\bar{z}}_{t}$ diverge slightly over time in our new formulation from what we found in our previous calculations.

Since the phenotypic variance is given by

$$\sigma_{z}^{2}=G_{\mathrm{aa}}\left(1+m\right)+G_{\mathrm{bb}}\varepsilon_{t-\tau }\left(\varepsilon_{t-\tau }+m\varepsilon_{t-\tau -1}\right)+\sigma_{e}^{2}+O\left(m^{2}\right)$$

and we have

$$\frac{\text{d(}\ln\bar{W)}}{\text{d}{\bar{z}}_{t}}=-\gamma \left({\bar{a}}_{t}-A+{\bar{b}}_{t}\varepsilon_{t-\tau }-B\varepsilon_{t}+m{\bar{z}}_{t-1}^{*}\right),$$

we also see that to $O\left(m\right)$ Equation (12) can be written

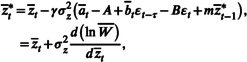

in agreement with Lande (1979) (eq. 6) and Kirkpatrick and Lande (1989) (eq. 2).

Similarly, under the weak selection or near equilibrium approximation, we can write:

$$\begin{array}{c} \Delta {\bar{a}}_{t}=C_{\mathrm{az}}\frac{d\left(\ln\bar{W}\right)}{d{\bar{z}}_{t}},\\ \Delta {\bar{b}}_{t}=C_{\mathrm{bz}}\frac{d\left(\ln\bar{W}\right)}{d{\bar{z}}_{t}}, \end{array}$$

where $C_{\mathrm{az}}$ and $C_{\mathrm{bz}}$ are the covariances of a and b with z, so our update equations for ${\bar{a}}_{t}$ and ${\bar{b}}_{t}$ also align with the formalism of Kirkpatrick and Lande (1989) (eq. 2).

Since updates to $\bar{e}$ only contribute to the dynamics through terms $m{\bar{z}}_{t-1}^{*}$ in the update equations for ${\bar{a}}_{t}$ and ${\bar{b}}_{t}$ it would also be legitimate to neglect them to first order in γ. We include them in our formalism here for consistency and to show the connection with other theoretical approaches.

**FIGURE B.1:**
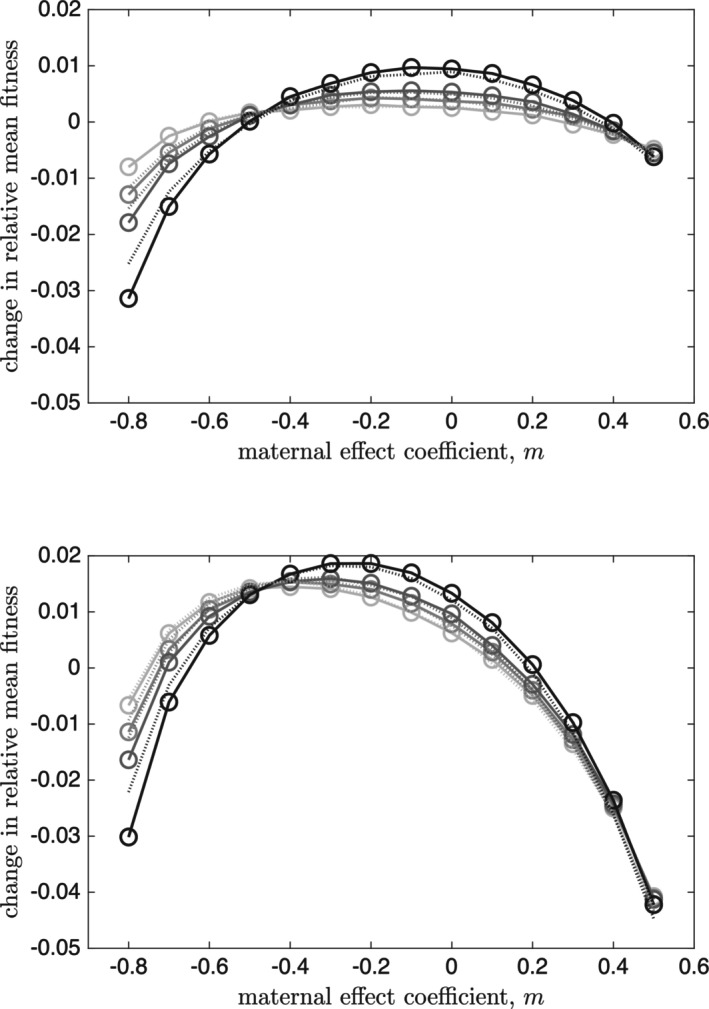
Relative differences in mean fitness value in the extended framework in stochastic environments in the noisy reference environment (top) and in a stochastic environment with δ=10 (bottom). Darker grey indicates larger strength of the environmental autocorrelation, $\rho_{\tau }$: From light to dark $\rho_{\tau }=\frac{1}{10}$, $\frac{1}{4}$, $\frac{1}{3}$, $\frac{1}{2}$, and fitness is maximised at −0.2, −0.2, −0.1 and −0.1 (top) and −0.4, −0.4, −0.3 and −0.2 (bottom), respectively. Model parameters follow Lande (2009), Hoyle and Ezard (2012): A=0, B=2, δ=10, τ=0.2, $\rho_{\tau }=0.25$, $\sigma_{e}^{2}=0.5$, $\sigma_{\xi }=2.0$, $G_{\mathrm{aa}}=0.5$, $G_{\mathrm{bb}}=0.045$, $\omega^{2}=50$ and $W_{\max}=1.0$. Circles represent the results of numerical simulations, where the arithmetic mean fitness is calculated over $10^{6}$ generations. The relative mean fitness is then calculated relative to the mean over m for each value of $\rho_{\tau }$. Results from a rerun of the original model (Hoyle and Ezard 2012) are shown as dotted lines for comparison.

**FIGURE B.2:**
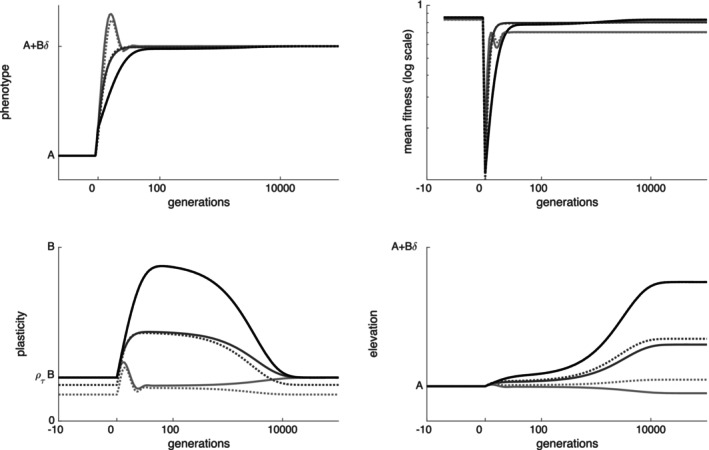
Evolution in the extended framework of the expected mean phenotype $E\left({\bar{z}}_{t}\right)$, expected mean fitness $E\left(\bar{W}\right)$ (plotted on a log scale), expected mean plasticity $E\left({\bar{b}}_{t}\right)$ and the expected mean elevation $E\left({\bar{a}}_{t}\right)$ in response to an environmental step change in the presence (dark grey, m=0.45; light grey, m=0.8) and absence (black, m=0) of a maternal effect with fixed coefficient m. Results for the original model (Hoyle and Ezard 2012) are shown as dotted lines for comparison. Parameters are as in Figure B.1.

**FIGURE B.3:**
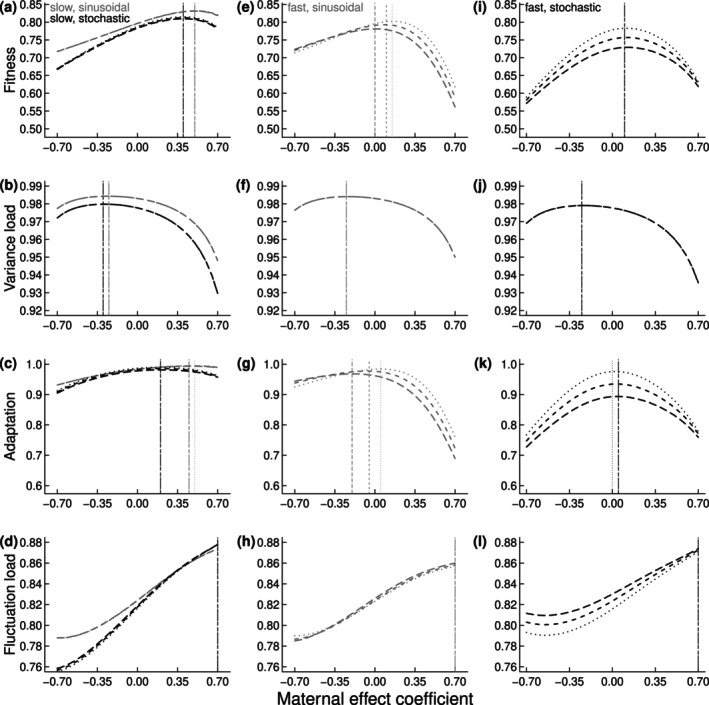
Time‐averaged expected mean fitness, variance load, adaptation and fluctuation load (Equations (B1), (B2), (B3)) for slow (left column) and fast (middle and right columns) stochastic (black) and sinusoidal (grey) environments with short, intermediate and long lags between juvenile development and adult selection (τ=0.05,0.20,0.35) in dotted, dashed and long‐dashed lines, respectively. The vertical lines indicate the values of the maternal effect coefficients that maximise each curve. Parameter values are A=0, B=2, $\rho_{\tau }=0.25$, $\sigma_{e}^{2}=0.5$, $\sigma_{\xi }=2$, $G_{\mathrm{aa}}=0.5$, $G_{\mathrm{bb}}=0.045$, γ=0.02, $\omega^{2}=50$ and $W_{\max}=1$.

**FIGURE B.4:**
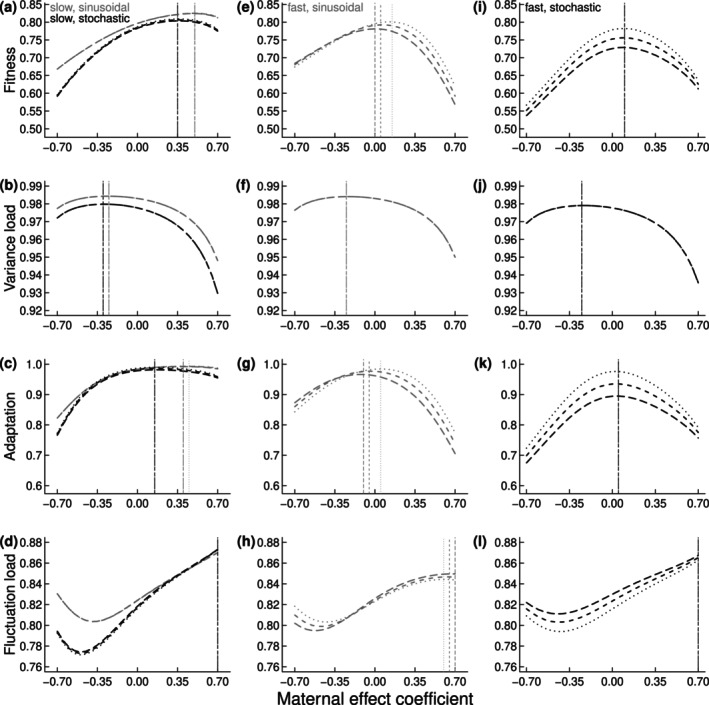
Time‐averaged expected mean fitness and components in the original framework of Ezard et al. (2014). Lines and parameters as in Figure B.3.

**FIGURE B.5:**
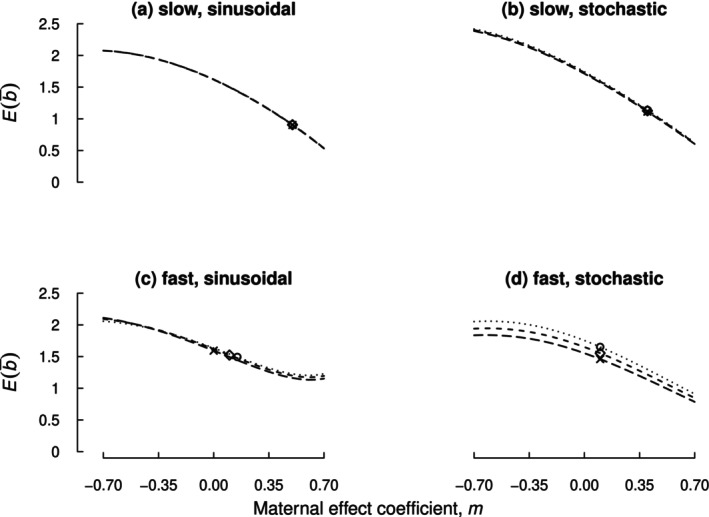
Time‐averaged expected values of the phenotypic plasticity slope ($E\left(\bar{b}\right)$) in the extended framework for slow (top), fast (bottom), sinusoidal (left) and stochastic (right) environments with short, intermediate and long lags between juvenile development and adult selection (τ=0.05,0.20,0.35) in dotted, dashed and long‐dashed lines, respectively. Points indicate the values of the components corresponding to the maternal effect coefficients that maximise expected mean fitness, with circles, diamonds and crosses for τ=0.05,0.20,0.35 respectively. Remaining parameter values are as in Figure B.3.

In Appendix B we repeat the main analyses of Hoyle and Ezard (2012) and Ezard et al. (2014) in the extended framework, incorporating the contributions from past states that we have set out above, and show that the results remain broadly qualitatively unchanged with typically only minor quantitative differences. Figures B.1 and B.2 reaffirm findings from Hoyle and Ezard (2012), notably that in a noisy equilibrium environment mean fitness is maximised at a negative value of m (Figure B.1), while during an environmental step change, positive fixed maternal effects speed up adaptation but lead to lower long‐term fitness (Figure B.2). Similarly Figures B.3 and B.5 confirm findings from Ezard et al. (2014) (see Figures B.4 and B.6) including the following. In more slowly changing environments, more strongly positive m maximises fitness and τ has no discernible impact (Figure B.3). In rapidly changing sinusoidal environments fitness is maximised for non‐negative m closer to zero as τ increases (Figure B.3). In rapidly flipping stochastic environments, larger τ leads to lower plasticity $E\left(\bar{b}\right)$ (Figure B.5) and fitness (Figure B.3) but does not affect the fitness‐maximising value of m (Figure B.3). In more rapidly changing environments, lower positive m and higher $E\left(\bar{b}\right)$ maximise fitness compared to the values for slowly changing environments (Figure B.5). A contrasting finding in the extended framework is that in slowly changing environments fluctuation load appears to have a greater effect than adaptation on fitness, whereas in the original model adaptation had a greater impact (Figure B.3).

## 4 Fixed Maternal and Grandmaternal Effects

Here we include fixed (evolutionarily constant) grandmaternal effects (Prizak et al. 2014) within our extended framework in addition to fixed maternal effects.

In order to keep track of the updates to ‘carried’ past states at selection in each generation, we now use the double star notation introduced in Section 2 such that $z_{t-2}^{**}$ represents the grandmaternal state after selection in both the grandmaternal and maternal generations. In contrast now $z_{t-2}^{*}$ represents the grandmaternal state after selection in the grandmaternal generation only.

Thus we write the phenotype as follows:

$$z_{t}=a_{t}+b_{t}\varepsilon_{t-\tau }+mz_{t-1}^{*}+gz_{t-2}^{**}+e_{t},$$

where g is the strength of the grandmaternal effect, which is assumed to be small in magnitude and of the same order as m.

The double star notation shows explicitly that we assume the grandmaternal state $z_{t-2}^{**}$ that appears in the expression for the current generation phenotype $z_{t}$ to be the state of the *maternal* grandmother, so that the maternal and grandmaternal genetic components are correlated. Prizak et al. (2014) made this assumption implicitly when calculating the phenotypic (co)variances, but in the extended framework it also impacts the form of the update equations.

We retain the contributions of the maternal and grandmaternal generations to the speed of evolution to first order in m and g, assuming that they are of the same order, giving

(13) $$\Delta {\bar{a}}_{t}\approx -\gamma G_{\mathrm{aa}}\left({\bar{a}}_{t}-A+{\bar{b}}_{t}\varepsilon_{t-\tau }-B\varepsilon_{t}+m{\bar{z}}_{t-1}^{*}+g{\bar{z}}_{t-2}^{**}\right)\left(1+\frac{m}{2}+\frac{g}{4}\right),$$

(14) $$\Delta {\bar{b}}_{t}\approx -\gamma G_{\mathrm{bb}}\left({\bar{a}}_{t}-A+{\bar{b}}_{t}\varepsilon_{t-\tau }-B\varepsilon_{t}+m{\bar{z}}_{t-1}^{*}+g{\bar{z}}_{t-2}^{**}\right)\left(\varepsilon_{t-\tau }+\frac{m}{2}\varepsilon_{t-\tau -1}+\frac{g}{4}\varepsilon_{t-\tau -2}\right),$$

(15)

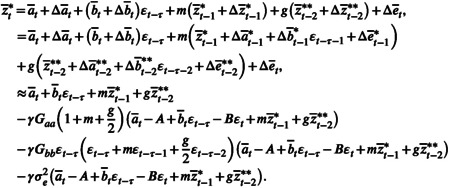




The update rule for ${\bar{z}}_{t}^{*}$ incorporates terms arising from the changes in distribution of the maternal and grandmaternal states and residuals at selection in the current generation given to leading order by

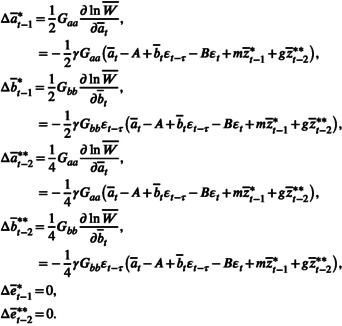




Neither $\Delta {\bar{e}}_{t-1}^{*}$ nor $\Delta {\bar{e}}_{t-2}^{**}$ have $O\left(1\right)$ terms and so they are both approximated to zero here.

Note that ${\bar{z}}_{t-2}^{**}$ is the average over the current generation of the grandmaternal phenotype, having passed through two rounds of selection (grandmaternal and maternal), while ${\bar{z}}_{t-2}^{*}$ as generated by Equation (15) in generation t−2 is the average over the *maternal* generation of the grandmaternal phenotype, having passed through only one round of selection (in the grandmaternal generation). Therefore to obtain ${\bar{z}}_{t-2}^{**}$ from ${\bar{z}}_{t-2}^{*}$ in the numerical simulations we must also take account of updates to the grandmaternal states at selection in the maternal generation:
(16)

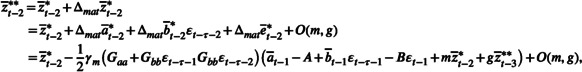

where we have used that

$$\begin{array}{c} \Delta_{\mathrm{mat}}{\bar{a}}_{t-2}^{*}=-\frac{1}{2}\gamma_{m}G_{\mathrm{aa}}\left({\bar{a}}_{t-1}-A+{\bar{b}}_{t-1}\varepsilon_{t-\tau -1}-B\varepsilon_{t-1}+m{\bar{z}}_{t-2}^{*}+g{\bar{z}}_{t-3}^{**}\right),\\ \Delta_{\mathrm{mat}}{\bar{b}}_{t-2}^{*}=-\frac{1}{2}\gamma_{m}G_{\mathrm{bb}}\varepsilon_{t-\tau -1}\left({\bar{a}}_{t-1}-A+{\bar{b}}_{t-1}\varepsilon_{t-\tau -1}-B\varepsilon_{t-1}+m{\bar{z}}_{t-2}^{*}+g{\bar{z}}_{t-3}^{**}\right),\\ \Delta_{\mathrm{mat}}{\bar{e}}_{t-2}^{*}=0, \end{array}$$

to $O\left(1\right)$ at selection in the maternal generation, and where $\gamma_{m}$ is the value of γ in the maternal generation. Note that we only need to calculate ${\bar{z}}_{t-2}^{**}$ to $O\left(1\right)$ since it only appears in the update equations for ${\bar{a}}_{t}$, ${\bar{b}}_{t}$ and ${\bar{z}}_{t}^{*}$ in the term $g{\bar{z}}_{t-2}^{**}$. Nonetheless as usual we retain the $O\left(m,g\right)$ terms in the $\left({\bar{z}}_{t-1}-\theta_{t-1}\right)$ bracket so as to preserve the common structure of the equations.

Similarly to the case of fixed maternal effects we see that to first order in m and g our update equations are equivalent to

$$\begin{array}{c} \Delta {\bar{a}}_{t}=C_{\mathrm{az}}\frac{d\left(\ln\bar{W}\right)}{d{\bar{z}}_{t}},\\ \Delta {\bar{b}}_{t}=C_{\mathrm{bz}}\frac{d\left(\ln\bar{W}\right)}{d{\bar{z}}_{t}},\\ \;{\bar{z}}_{t}^{*}={\bar{z}}_{t}+\sigma_{z}^{2}\frac{d\left(\ln\bar{W}\right)}{d{\bar{z}}_{t}}, \end{array}$$

where we have calculated

$$\sigma_{z}^{2}=\left(1+m+\frac{g}{2}\right)G_{\mathrm{aa}}+\epsilon_{t-\tau }\left(\epsilon_{t-\tau }+m\epsilon_{t-\tau -1}+\frac{g}{2}\epsilon_{t-\tau -2}\right)G_{\mathrm{bb}}+\sigma_{e}^{2}$$

to first order. Again this shows that our formalism aligns with that of Kirkpatrick and Lande (1989) (eq. 2).

**FIGURE C.1:**
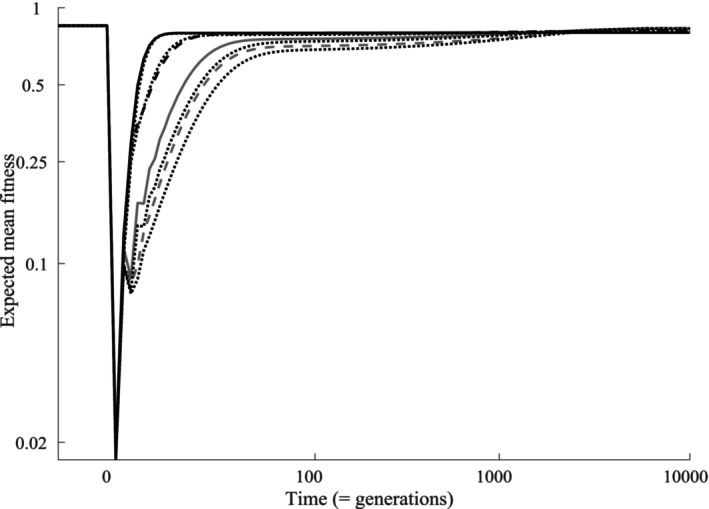
Evolution of the expected mean fitness during adaptation to an extraordinary new environment when fixed grandmaternal and maternal effects are included in the extended framework. The expected mean fitness is plotted on a logarithmic scale with $W_{\max}=1$, for all four combinations of m±0.35 and g±0.15. Black (grey) lines show the m>0 (m<0) case; solid (dashed) lines show the g>0 (g<0) case. The parameter values follow Lande (2009), Hoyle and Ezard (2012), Prizak et al. (2014): A=0, B=2, $\rho_{\tau }=0.25$, $\sigma_{e}^{2}=0.5$, $\sigma_{\xi }=2$, $G_{\mathrm{aa}}=0.5$, $G_{\mathrm{bb}}=0.045$, $\omega^{2}=50$ and δ=10. Results in the original (Prizak et al. 2014) framework are shown with dotted lines for comparison.

**FIGURE C.2:**
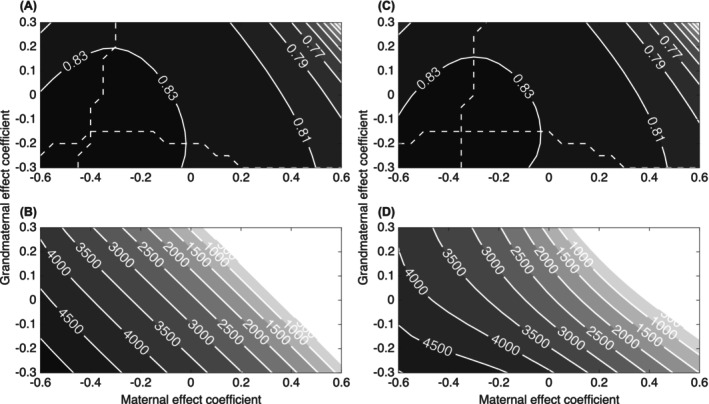
Values in the extended framework with fixed grandmaternal and maternal effects for (A) the expected mean fitness after 100,000 generations and (B) number of generations to recover equilibrium fitness after the environmental step change, defined as returning to and remaining within 0.01 of the fitness attained 100,000 generations after the step change. Panels (C) and (D) show the same quantities in the corrected original framework of Prizak et al. (2014) for comparison. The values of the expected mean fitness are very similar in both frameworks, while the return to equilibrium is a little faster in the extended framework over most of the domain. The dotted lines in panels (A) and (C) show the maximum expected mean fitness (within the domain −0.6≤m≤0.6, −0.3≤g≤0.3) holding either maternal or grandmaternal effects constant. Parameter values as in Figure C.1.

In Appendix C we repeat the analyses of Prizak et al. (2014) in the extended framework and show that once again the results remain largely qualitatively unchanged between the original and extended framework models with typically only minor quantitative differences. This is because the expected fitness at equilibrium is largely determined by the expected phenotypic variance which is unchanged in the extended framework, and the differences in the speed of evolution are small. In both the extended framework and original models we see that recovery of fitness after an environmental step change is faster for positive compared to negative m, and for positive compared to negative g (Figures C.1 and C.2B), and that the fitness‐maximising values of m and g are both negative (Figure C.2A).

## 5 Evolving Maternal Effects

### 5.1 Evolution Equations

Now we consider the case where maternal effects, m, can evolve as in Kuijper and Hoyle (2015) so that the current generation genetic components evolve according to

$$\begin{array}{c} \left(\begin{array}{c} \Delta {\bar{a}}_{t}\\ \Delta {\bar{b}}_{t}\\ \Delta {\bar{m}}_{t} \end{array}\right)=\left(\begin{array}{c} G_{\mathrm{aa}}\left(\frac{\partial }{\partial {\bar{a}}_{t}}+\sum_{n=1}^{\infty }\frac{1}{2^{n}}\frac{\partial }{\partial {\bar{a}}_{t-n}^{*\cdots *}}\right)\\ G_{\mathrm{bb}}\left(\frac{\partial }{\partial {\bar{b}}_{t}}+\sum_{n=1}^{\infty }\frac{1}{2^{n}}\frac{\partial }{\partial {\bar{b}}_{t-n}^{*\cdots *}}\right)\\ G_{\mathrm{mm}}\left(\frac{\partial }{\partial {\bar{m}}_{t}}+\sum_{n=1}^{\infty }\frac{1}{2^{n}}\frac{\partial }{\partial {\bar{m}}_{t-n}^{*\cdots *}}\right) \end{array}\right)\ln\bar{W}. \end{array}$$

We assume throughout that $a_{t}$, $b_{t}$, $m_{t}$, $z_{t-1}^{*}$ and $z_{t}$ are normally distributed, but note that in the case of $z_{t-1}^{*}$ and $z_{t}$ this can only be an approximation or heuristic, since

$$z_{t}=a_{t}+b_{t}\varepsilon_{t-\tau }+m_{t}z_{t-1}^{*}+e_{t}$$

is constructed as the sum of terms including the product of a normally distributed variable $m_{t}$ with an approximately normally distributed variable $z_{t-1}^{*}$, which in general is not normal. This is a limitation of both our original and extended framework models when $m_{t}$ is distributed across the population rather than taking a fixed value. It means that the expression for the mean fitness (Equation 17 below; eq. 3 of Kuijper and Hoyle (2015)) is an approximation or heuristic (in addition to the weak selection approximation shown in the equation). When $\left|m_{t}\right|$ and $G_{mm}$ are small, the product term $m_{t}z_{t-1}^{*}$ that leads to non‐normality of $z_{t}$ is also small and so we would expect the approximation to be reasonable, but we have not attempted to assess its impact. In Kuijper and Hoyle (2015) the calculations in the phenotypic variance section of the Appendix also depend on $z_{t-1}^{*}$ being multivariate normal with $a_{t}$, $b_{t}$ and $m_{t}$, but for the equivalent calculations in the extended framework it is enough for $a_{t}$, $b_{t}$, $m_{t}$, $e_{t}$ and their past states to be multivariate normal.

Following Kuijper and Hoyle (2015), individual fitness is given by

$$\begin{array}{c} W\left(z_{t},b_{t},m_{t}\right)=W_{\max}\exp\left[-\frac{{\left(z_{t}-\theta_{t}\right)}^{2}}{2\omega_{z}^{2}}-\frac{b_{t}^{2}}{2\omega_{b}^{2}}-\frac{m_{t}^{2}}{2\omega_{m}^{2}}\right], \end{array}$$

where the width of the fitness function is now denoted $\omega_{z}$, where $\omega_{b}$ is an inverse measure of the cost of within‐generation phenotypic plasticity, and where $\omega_{m}$ is an inverse measure of the cost of maternal effects. The population mean fitness is given by

$$\begin{array}{c} {\bar{W}}_{t}\approx W_{\max}\sqrt{\gamma_{z}\gamma_{b}\gamma_{m}\omega_{z}^{2}\omega_{b}^{2}\omega_{m}^{2}}\exp\left\{-\frac{1}{2}\left(\gamma_{z}{\left({\bar{z}}_{t}-\theta_{t}\right)}^{2}+\gamma_{b}{\bar{b}}_{t}^{2}+\gamma_{m}{\bar{m}}_{t}^{2}\right)\right\}+O\left(\frac{1}{\omega^{4}}\right), \end{array}$$

where $\gamma_{z}=1/\left(\omega_{z}^{2}+\sigma_{z_{t}}^{2}\right)$, $\gamma_{b}=1/\left(\omega_{b}^{2}+G_{\mathrm{bb}}\right)$ and $\gamma_{m}=1/\left(\omega_{m}^{2}+G_{\mathrm{mm}}\right)$. Here ω represents any of $\omega_{z}$, $\omega_{b}$ and $\omega_{m}$, and for weak selection $1/\omega^{2}$ is assumed to be small.

Since in the extended framework we have an infinite sum of terms in the update equations, we will need to make an approximation in order to make progress, and the simplest approximation is to keep leading order terms in m (where here m represents any of its versions in any generation). In that case the update equations for the current generation genetic components (eq. 8a–c of Kuijper and Hoyle (2015)) and the maternal genetic components become

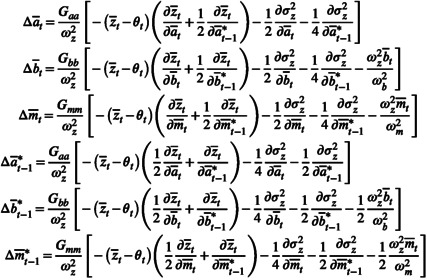

to the first two leading orders in m and leading order in $1/\omega^{2}$. We also add the updates to $\bar{e}$:

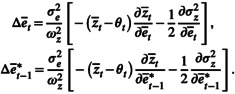




The population mean phenotype is given by

$${\bar{z}}_{t}={\bar{a}}_{t}+{\bar{b}}_{t}\varepsilon_{t-\tau }+\bar{m_{t}z_{t-1}^{*}}+{\bar{e}}_{t},$$

where ${\bar{e}}_{t}=0$ and the phenotypic variance is 
(18)

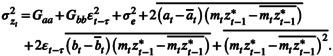




In contrast to the cases of fixed maternal and grandmaternal effects, the phenotypic variance is not constant, and so its derivatives now contribute to the update equations.

We show in Appendix D that we can use these expressions to derive the update equations for the current states in the form

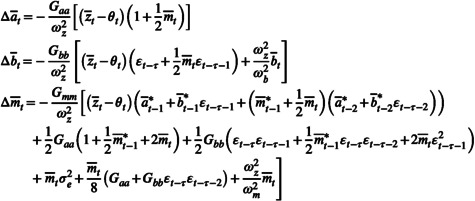

to $O\left(m/\omega^{2}\right)$ for ${\bar{a}}_{t}$ and ${\bar{b}}_{t}$ and to $O\left(m^{3}/\omega^{2}\right)$ for ${\bar{m}}_{t}$, where we assume that $G_{\mathrm{mm}}∼O\left(m^{2}\right)$. We retain terms at the two leading orders for each equation, and also retain implicit $O\left(m^{2}\right)$ terms in products involving $\left({\bar{z}}_{t}-\theta_{t}\right)$ so as to retain the functional structure of the equations.

The update equations can also be written as follows:

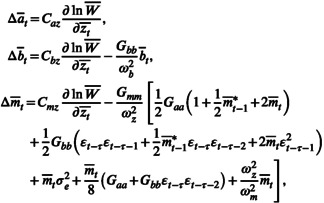

to the same order of approximation. These align with the expected Kirkpatrick and Lande (1989) expressions, $C_{\mathrm{xz}}\frac{\partial \ln\bar{W}}{\partial {\bar{z}}_{t}}$, where x=a,b,m, with additions arising from: (i) the costs of within‐generation plasticity and maternal effects, and (ii) the dependence of the phenotypic variance on ${\bar{m}}_{t}$ and ${\bar{m}}_{t}^{*}$ owing to the non‐normality of $z_{t}$ and the use of the heuristic expression for mean fitness (Equation 17).

We can use the equalities ${\bar{a}}_{t-1}^{*}={\bar{a}}_{t}$, ${\bar{b}}_{t-1}^{*}={\bar{b}}_{t}$ and ${\bar{m}}_{t-1}^{*}={\bar{m}}_{t}$ to simplify further as required. This is implemented in the simulation code, both in the update equation for ${\bar{m}}_{t}$ which becomes

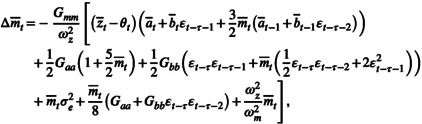

and in the expressions for the mean phenotype and the phenotypic variance, which we approximate as follows:
(19)

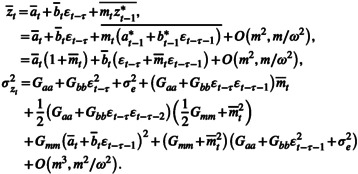




By making a small m approximation we have been able to express all required quantities in terms of ${\bar{a}}_{t}$, ${\bar{b}}_{t}$, ${\bar{m}}_{t}$, ${\bar{a}}_{t-1}$, ${\bar{b}}_{t-1}$ and so in contrast to Kuijper and Hoyle (2015) no complicated calculations are required to update the variance or covariances. However, the small m approximation is of course only valid when ∣m∣ is small enough. In particular we are effectively making the approximation $\left(1-G_{\mathrm{mm}}-{\bar{m}}_{t}^{2}\right)\approx 1$ in a factor that appears in the denominator of the expression for the phenotypic variance in Kuijper and Hoyle (2015), and so the approximation is no longer a good one when $|{\bar{m}}_{t}|$ approaches 1. We will explore this further in Section 5.2 below.

A further limitation of the model is that for consistency in the order of expansion of the approximations ∣m∣ should strictly be larger than $1/\omega^{2}$. This assumption is violated when m is very close to zero, which can and does happen during the simulations as m evolves. However we would expect any errors arising from this to be very small.

Equilibrium solutions in a constant environment $\varepsilon_{t}=\varepsilon$ satisfy

$$\begin{array}{c} \bar{z}=\theta ,\bar{b}=0,⟹\\ \bar{a}=\left(1-\bar{m}\right)\left(A+\mathrm{B\varepsilon}\right),\\ \bar{m}=-{\left\{\frac{11}{4}+\frac{2\left(\sigma_{e}^{2}+\omega_{z}^{2}/\omega_{m}^{2}\right)}{\left(G_{\mathrm{aa}}+G_{\mathrm{bb}}\varepsilon^{2}\right)}\right\}}^{-1}, \end{array}$$

and so the equilibrium solution has a negative mean maternal effect coefficient (as was also the case in Kuijper and Hoyle (2015)).

The initial development of maternal effects from an equilibrium state ${\bar{z}}_{t}=\theta$ in a constant environment ε, on allowing for a small genetic variation in maternal effects $G_{\mathrm{mm}}$, is given by

$${\left.\Delta {\bar{m}}_{t}\right|}_{{\bar{m}}_{t}=0,{\bar{z}}_{t}=\theta }=-\frac{G_{\mathrm{mm}}}{2\omega_{z}^{2}}\left(G_{\mathrm{aa}}+G_{\mathrm{bb}}\varepsilon^{2}\right)$$

to leading order and so the initial evolution will be towards negative values, confirming what was deduced from eq. (9) of Kuijper and Hoyle (2015).

**FIGURE 1:**
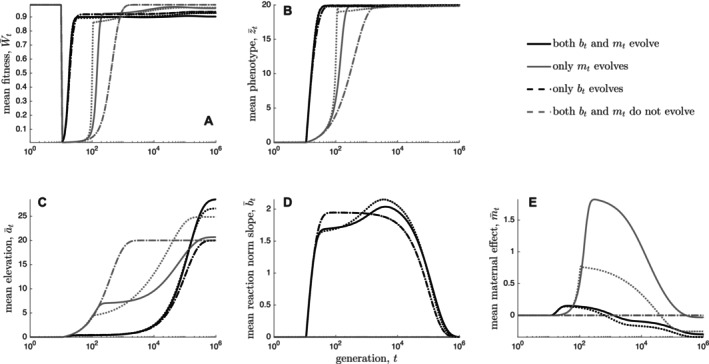
Numerical simulations showing the evolutionary response to a sudden shift in the environment $\varepsilon_{t}$ at t=10 in the extended framework model for evolving maternal effects, where $\varepsilon_{t}=\delta U_{t-10}+\xi_{t}$, $U_{t-10}$ is the unit step function, δ is the amplitude of the shift, and $\xi_{t}$ is additive noise as in Appendix B. Here we allow each of within‐generational plasticity, ${\bar{b}}_{t}$, and maternal effects, ${\bar{m}}_{t}$, to evolve or force them to remain constant, and show the course of evolution for all four possibilities: Both $b_{t}$ and $m_{t}$ evolve (solid black lines); only $m_{t}$ evolves (solid grey lines); only $b_{t}$ evolves (dashed black lines); neither $b_{t}$ nor $m_{t}$ evolve (dashed grey lines). Panels show (A) population mean fitness ${\bar{W}}_{t}$, (B) mean phenotype ${\bar{z}}_{t}$, (C) mean elevation ${\bar{a}}_{t}$, (D) mean slope of plasticity reaction norm ${\bar{b}}_{t}$ and (E) mean maternal effect coefficient ${\bar{m}}_{t}$. Results from Kuijper and Hoyle (2015) are plotted as dotted lines for comparison. Where a line appears to be dot‐dashed that is because the results in the extended framework and those of Kuijper and Hoyle (2015) lie very close to each other. Model parameters used: $G_{\mathrm{aa}}=0.1,G_{\mathrm{bb}}=0.045,G_{\mathrm{mm}}=0.005,\omega_{z}^{2}=40,A=0,B=2,\sigma_{\xi }^{2}=0.0001,\rho_{\tau }=0.5,\delta =10,\tau =0.25,\sigma_{e}^{2}=1,\omega_{b}^{2}=\omega_{m}^{2}=100,W_{\max}=1.$ (When $b_{t}$ or $m_{t}$ is prevented from evolving $G_{\mathrm{bb}}$ or $G_{\mathrm{mm}}$ respectively is set to zero.)

**FIGURE 2:**
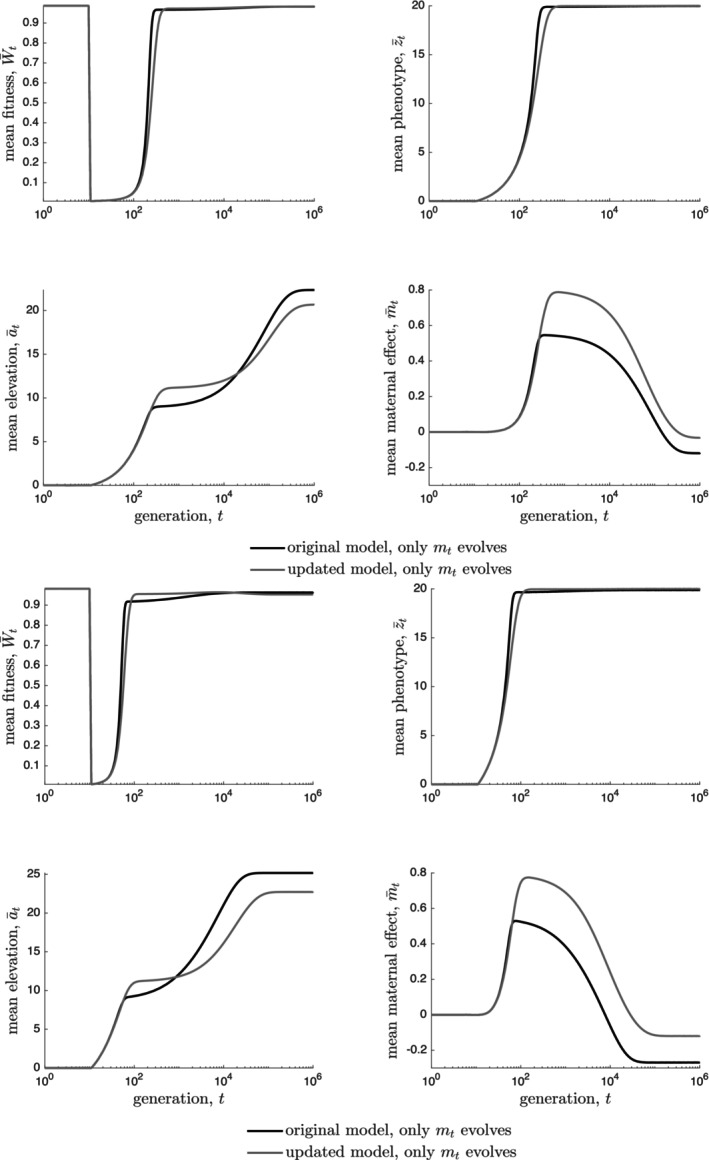
Comparison between the original Kuijper and Hoyle (2015) model and the revised model using a small m approximation, for evolution in response to a sudden shift in the environment, when either $G_{\mathrm{mm}}$ is small ($G_{\mathrm{mm}}=0.001$, Top Panels) or $G_{\mathrm{aa}}$ is large ($G_{\mathrm{aa}}=0.5$, Bottom Panels) in the case when the maternal effect evolves and within‐generation plasticity does not. All other parameters are as given in Figure 1.

Numerical simulations of the evolution during rapid environmental change are shown in Figure 1 and compared to trajectories from the original model (dotted lines; see also Kuijper and Hoyle 2015, Fig. 2), with both models showing ${\bar{m}}_{t}$ evolving to positive values for many generations following the environmental shift before gradually declining to negative values after a long time. The results are very similar, except when within‐generation plasticity, $b_{t}$, does not evolve, in which case while behaviour remains qualitatively similar to the original, ${\bar{m}}_{t}$ grows larger, quickly becoming too large for the small m approximation to hold. This effect can be reduced by setting $G_{\mathrm{aa}}$ large enough or $G_{\mathrm{mm}}$ small enough that the phenotype grows close to the optimum before ${\bar{m}}_{t}$ grows too large (Figure 2). In other words the faithfulness of our $O\left(m\right)$ approximation depends on whether we are working in a parameter regime in which our small m assumption holds, that is, $|{\bar{m}}_{t}|$ remains small, over the full period under consideration.

**FIGURE 3:**
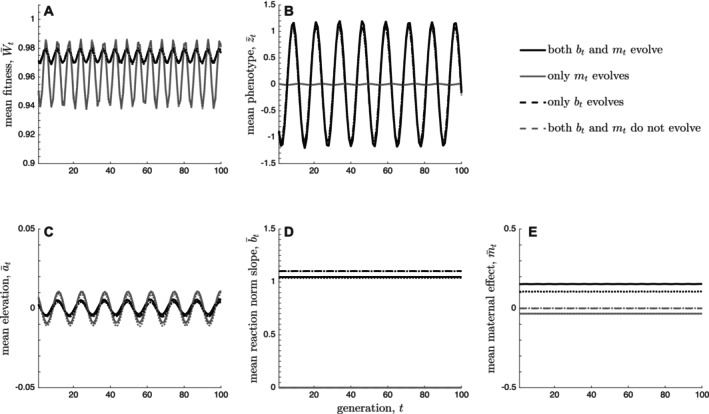
Numerical simulations showing adaptation to a sinusoidal environment, $\varepsilon_{t}=\sin ft+\xi_{t}$, in the extended framework with evolving maternal effects, where $\xi_{t}$ is additive noise as in Appendix B. Panels as in Figure 1. Results from the Kuijper and Hoyle (2015) model are shown as dotted lines for comparison. The model parameters are $G_{\mathrm{aa}}=0.1$, $G_{\mathrm{bb}}=G_{\mathrm{mm}}=0.045$, $\omega_{z}^{2}=40$, A=0, B=2, $\sigma_{\xi }^{2}=0.0001$, $\rho_{\tau }=0.5$, τ=0.25, $\sigma_{e}^{2}=1$, $\omega_{m}^{2}=\omega_{b}^{2}=100$, $W_{\max}=1$. The amplitude of the sine wave is 1 and its frequency is f=0.5.

**FIGURE 4:**
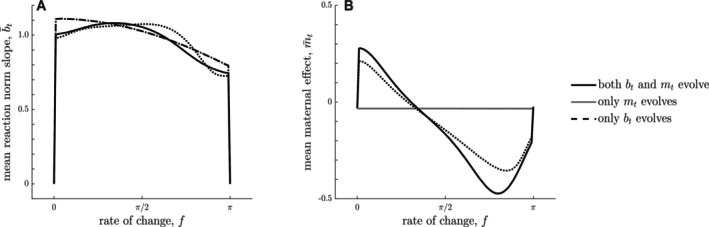
Evolved values of (A) mean plasticity reaction norm slope $\bar{b}$ and (B) mean maternal effect coefficient $\bar{m}$ with varying frequency of environmental change f. Results from the Kuijper and Hoyle (2015) model are shown as dotted lines for comparison. Parameters as in Figure 3.

**FIGURE 5:**
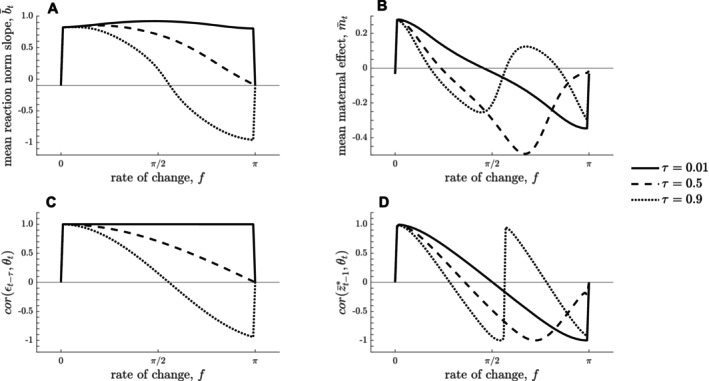
Evolved values of (A) mean plasticity reaction norm slope $\bar{b}$ and (B) mean maternal effect coefficient $\bar{m}$, and correlations (C) $\mathrm{cor}\left(\varepsilon_{t-\tau },\theta_{t}\right)$ and (D) $\mathrm{cor}\left({\bar{z}}_{t-1}^{*},\theta_{t}\right)$ with varying frequency of environmental change f, and for various values of τ, the lag between development and selection. Parameters as in Figure 3 except $\sigma_{\xi }^{2}=0$. Note that the correlations are not defined at f=0,π, since in that case $\theta_{t}=0$ for all t, and we choose to plot these points as zero motivated by the corresponding values of ${\bar{b}}_{t}$ and ${\bar{m}}_{t}$.

The evolutionary response to a sinusoidal environment shows qualitatively very similar behaviour to the original model (Figure 3), except that at the parameter values investigated $|{\bar{m}}_{t}|$ can grow somewhat larger than in the original (Figures 3E and 4B) and ${\bar{b}}_{t}$ can be somewhat smaller (Figure 4A) when ${\bar{m}}_{t}$ and ${\bar{b}}_{t}$ both evolve. The values of ${\bar{b}}_{t}$ and ${\bar{m}}_{t}$ can still be understood as largely following the form of $\mathrm{cor}\left(\varepsilon_{t-\tau },\theta_{t}\right)$ and $\mathrm{cor}\left({\bar{z}}_{t-1}^{*},\theta_{t}\right)$ respectively (Figure 5). To plot Figure 5D, we constructed ${\bar{z}}_{t-1}^{*}$ using the approximation

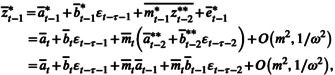

and noting that ${\bar{e}}_{t-1}^{*}∼O\left(1/\omega^{2}\right)$, ${\bar{a}}_{t-2}^{**}={\bar{a}}_{t-2}^{*}+O\left(1/\omega^{2}\right)$ and ${\bar{b}}_{t-2}^{**}={\bar{b}}_{t-2}^{*}+O\left(1/\omega^{2}\right)$.

### 5.2 Heuristic Method to Limit the Growth of ${\bar{m}}_{t}$ When the Phenotypic Variance Diverges

As described above, the small m approximation that we employ can only be used when ∣m∣<1. It is a good approximation when ∣m∣≪1, and an increasingly poor approximation as ∣m∣ approaches 1. When m is fixed, we can handle this by only considering values of m for which the approximation is reasonable. However, when $m_{t}$ is allowed to evolve, the approximate evolution equations can lead ∣m∣ to grow big enough that the approximation is no longer so good, or even becomes invalid—see for example Figure 1E, where ${\bar{m}}_{t}$ grows transiently larger than 1 in the case where only $m_{t}$ evolves. A likely reason for this is that, as mentioned above, we have approximated $\left(1-G_{\mathrm{mm}}-{\bar{m}}_{t}^{2}\right)\approx 1$ in the denominator of the expression for the phenotypic variance, and that means the variance no longer grows very large as ${\bar{m}}_{t}^{2}$ approaches 1 (or strictly $1-G_{\mathrm{mm}}$), and so no longer damps down the growth of $|{\bar{m}}_{t}|$ as it otherwise would have done. In a formal approximation of the evolution equations, there is nothing we can do about this other than to note that our approximation breaks down if $|{\bar{m}}_{t}|$ grows too large. However, if we are willing to take a more heuristic (pragmatic) approach, then we can reintroduce the factor in the denominator of the phenotypic variance that keeps $|{\bar{m}}_{t}|$ from growing too large, so that the approximation used in the rest of the evolution equations remains valid.

To implement this heuristic method for preventing $|{\bar{m}}_{t}|$ from growing too large we first notice that the term $\left(G_{\mathrm{aa}}+G_{\mathrm{bb}}\varepsilon_{t-\tau -1}^{2}+\sigma_{e}^{2}\right)$ in the phenotypic variance (Equation 19) is the leading order approximation to the term $\sigma_{z_{t-1}^{*}}^{2}$ appearing in the expansion of the final term in Equation (18). Making a weak selection (close to equilibrium) approximation we can write $\sigma_{z_{t-1}^{*}}^{2}\approx \sigma_{\mathrm{zt}}^{2}$, so that we have

(20) $$\begin{array}{c} \begin{array}{c} \sigma_{z_{t}}^{2}\approx \frac{1}{1-G_{\mathrm{mm}}-{\bar{m}}_{t}^{2}}\left\{G_{\mathrm{aa}}+G_{\mathrm{bb}}\varepsilon_{t-\tau }^{2}+\sigma_{e}^{2}+\left(G_{\mathrm{aa}}+G_{\mathrm{bb}}\varepsilon_{t-\tau }\varepsilon_{t-\tau -1}\right){\bar{m}}_{t}\right.\\ \;+\frac{1}{2}\left(G_{\mathrm{aa}}+G_{\mathrm{bb}}\varepsilon_{t-\tau }\varepsilon_{t-\tau -2}\right)\left(\frac{1}{2}G_{\mathrm{mm}}+{\bar{m}}_{t}{\bar{m}}_{t-1}^{*}\right)\\ \;+G_{\mathrm{mm}}\left.{\left({\bar{a}}_{t-1}^{*}+{\bar{b}}_{t-1}^{*}\varepsilon_{t-\tau -1}\right)}^{2}\right\}, \end{array} \end{array}$$

a heuristic expression that nearly matches Equation (19) to $O\left(m^{2}\right)$ for small ∣m∣ and $1/\omega^{2}$ (except that $\varepsilon_{t-\tau -1}^{2}$ in the last term of Equation (19) is replaced by $\varepsilon_{t-\tau }^{2}$), but diverges for larger ∣m∣. (Note that the heuristic will match less well if the environment changes rapidly between successive generations, owing to the difference between $\varepsilon_{t-\tau -1}^{2}$ and $\varepsilon_{t-\tau }^{2}$, but this only happens for a single timestep at the step change in our simulations and so does not have a significant impact on the dynamics).

In this case, as shown in Appendix D, the update equation for ${\bar{m}}_{t}$ becomes

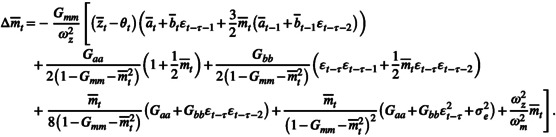




**FIGURE 6:**
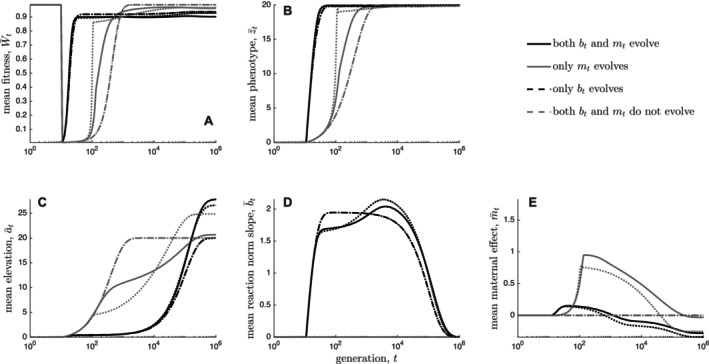
Evolution in the extended framework using the heuristic method of controlling the growth of ${\bar{m}}_{t}$ described in Section 5.2. Panels and parameters as in Figure 1.

Using this heuristic, $|{\bar{m}}_{t}|$ is prevented by the divergence of the phenotypic variance from growing large in the case when within‐generation plasticity, $b_{t}$, does not evolve and remains much closer to the values seen in the original model, as can be seen in Figure 6. (We know that the results of the original model are themselves approximately correct because they were validated with individual‐based modelling in Kuijper and Hoyle (2015)). In the case where only ${\bar{m}}_{t}$ evolves it grows to a value close to 1, before declining, similar to what is seen in the individual‐based simulations in Fig. S1 of Kuijper and Hoyle (2015).

## 6 Discussion

Our extended theoretical framework for cascading maternal effects makes clear how the changing distribution of past maternal lineage states in the current population affects the selection of genetic components and phenotypes in the current generation, and at the same time explicitly aligns our approach with that of Kirkpatrick and Lande (1989).

In the cases we have investigated here the extended framework typically leads to small quantitative changes in the evolution of the genetic components and phenotype, but qualitatively the results remain very similar. The principal exception to this is when evolving maternal effects grow large in the absence of within‐generational phenotypic plasticity, a scenario that could potentially be relevant in situations such as lecithotrophy (Vance 1973; Strathmann 1985; Marshall et al. 2008). However, this can largely be controlled by a heuristic adjustment to the evolution equation for the maternal effect coefficient.

While the extended framework is conceptually more complex than our original, the necessity of restricting attention to small maternal effect values and making a corresponding approximation to the update equations leads to a simpler analysis of evolving maternal effects that may facilitate further research. Furthermore, its comprehensiveness provides a clearer interpretation of the evolution of the different contributions to the phenotype. The extended framework also opens the way to analysing more complex patterns of cascading transgenerational effects by integrating the constantly updating history of past maternal genetic components within a single flexible framework, and offers the possibility of a further extension to include the history of paternal genetic components.

## Author Contributions

**Rebecca B. Hoyle:** conceptualization (lead), formal analysis (lead), software (lead), visualization (lead), writing – original draft (lead), writing – review and editing (equal). **Thomas H. G. Ezard:** conceptualization (supporting), writing – review and editing (equal). **Bram Kuijper:** conceptualization (supporting), writing – review and editing (equal).

## Funding

The authors declare no relevant funding.

## Conflicts of Interest

The authors declare no conflicts of interest.

## Figures and Tables

**FIGURE B.6:**
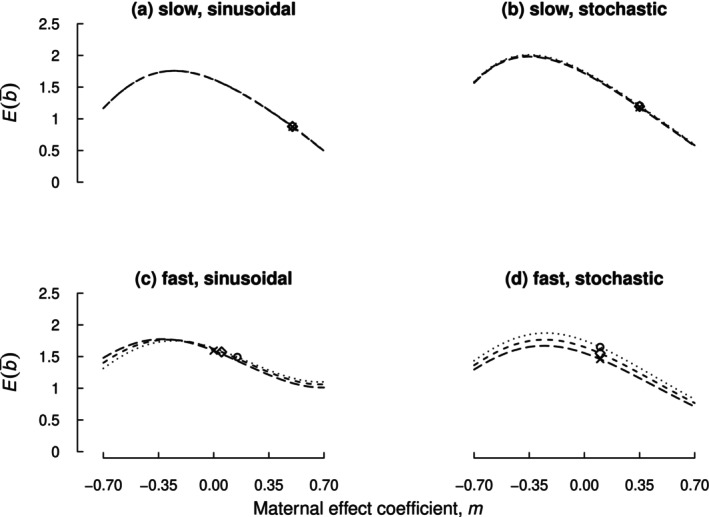
Time‐averaged values of the phenotypic plasticity slope ($E\left(\bar{b}\right)$) in the original framework of Ezard et al. (2014). Lines and parameters as in Figure B.5.

## Data Availability

The code for running the numerical simulations and producing the figures can be found at https://doi.org/10.5281/zenodo.17988677.

## Appendix A Updates to Past Maternal States

Updates at each generation take place in two stages: (1) parents produce successful offspring in the next generation according to their own fitness, and (2) the offspring genetic values and past maternal values are derived from the parents' genetic values and past maternal values. Lande (1979) rolls these updates into one step in Equation (4). However the current genetic components and past states in our framework are passed on differently to the offspring: current genetic components are inherited sexually, while the history of past states is passed on solely through the mothers. Thus, we need to be sure that Equation (4) holds in all cases.

First we consider the genetic components a, b and m that are inherited sexually with mating at random. The mean genetic values in the next generation are calculated by considering the distribution of genes over all parents. Using the genetic component a as an example, we have

$$\begin{array}{c} {\bar{a}}_{t+1}=\frac{1}{\bar{W}}\int_{\mathrm{all}\;\text{parents}}a_{t}g\left(x\right)\tilde{W}\left(x\right)dx. \end{array}$$

Assuming that the vectors of genetic component history x (that include both current and past maternal states) are identically distributed in mothers and fathers, and that the mean fitness of a given genetic component history is the same in mothers and fathers, then the relationship between the offspring and post‐selection maternal genetic values is given by

(A1)
$$\begin{array}{c} {\bar{a}}_{t+1}=\frac{1}{\bar{W}}\int_{\mathrm{all}\;\text{parents}}a_{t}g\left(x\right)\tilde{W}\left(x\right)dx\\ \;=\frac{1}{\bar{W}}\int_{\text{mothers}}a_{t}g\left(x\right)\tilde{W}\left(x\right)dx={\bar{a}}_{t}^{*}. \end{array}$$

Now considering updates to past states that are passed on only by mothers, their mean values in generation t+1 are given by

$${\left({\bar{a}}_{t-n}^{*\ldots *}\right)}^{*}=\frac{1}{\bar{W}}\int_{\text{mothers}}a_{t-n}^{*\ldots *}g\left(x\right)\tilde{W}\left(x\right)dx=\frac{1}{\bar{W}}\int_{\mathrm{all}\;\text{parents}}a_{t-n}^{*\ldots *}g\left(x\right)\tilde{W}\left(x\right)dx,$$

where the second equality holds because genetic component histories are identically distributed in fathers and mothers, and the mean fitness of a given genetic component history is the same in both. Thus, even though the two inheritance mechanisms are different, Equation (4) holds in both cases.

If we now explicitly label x with the generation t in the integral in Equation (A1) it is possible to see that the * denoting the maternal genetic component after selection indicates that the population mean of the maternal genetic value is taken over the distribution in the offspring generation and that when integrating over the maternal generation the fitness function $W\left(x_{t}\right)$ weights the maternal genetic components in proportion to the mean number of successful offspring that mothers with that genetic component history produce:

$${\bar{a}}_{t}^{*}=\frac{1}{\bar{W}}\int_{\text{mothers}}a_{t}g\left(x_{t}\right)\tilde{W}\left(x_{t}\right)dx_{t}=\int a_{t}^{*}g\left(x_{t+1}\right)dx_{t+1}$$

Thus whereas in Ezard et al. (2014) and Prizak et al. (2014) we stated that

$${\bar{a}}_{t}={\bar{a}}_{t-1}^{*},\;{\bar{b}}_{t}={\bar{b}}_{t-1}^{*},\;{\bar{m}}_{t}={\bar{m}}_{t-1}^{*}$$

were not true for fertility selection, it is now clear that because the population mean is taken over the distribution of states in the current generation, the maternal genetic component from each mother is weighted in proportion to the number of her offspring in the current generation, and so these relationships hold true for both viability and fertility selection. (See also Hadfield (2012).) Thus our models are more general than we previously stated.

While we have assumed that the distribution of the genetic component history, x, and the mean fitness of a given genetic component history are the same in males and females, it would be interesting to relax these assumptions and study the consequences of sex differences in selection (see for example Lande (1980)).

## Appendix B Calculations for Fixed Maternal Effects

Here we repeat the main analyses of Hoyle and Ezard (2012) and Ezard et al. (2014) within the extended framework.

### Fitness in a Noisy Equilibrium Environment

Following Hoyle and Ezard (2012) we consider the mean fitness of a population evolving in a noisy equilibrium environment $\varepsilon_{t}=\delta +\xi_{t}$, where δ is a constant and $\xi_{t}$ is a Gaussian stationary autocorrelated random process with mean zero, variance $\sigma_{\xi }^{2}$ and autocorrelation $\rho_{\tau }$ over the interval τ.

We simulate the evolution numerically using Equations ((10), (11), (12)). The results (Figure B.1) remain both qualitatively and quantitatively very close to those of Hoyle and Ezard (2012), with mean fitness maximised at negative maternal effect coefficient m, a trend of the fitness‐maximising value of m becoming less negative as the environment becomes more predictable (higher $\rho_{\tau }$) and greater relative fitness costs in the δ=10 environment, compared to the noisy reference environment (δ=0). A minor difference is that in the noisy reference environment none of the values of $\rho_{\tau }$ provide a sufficiently predictable environment for m=0 to maximise fitness in the extended framework, whereas in Hoyle and Ezard (2012) this was the case for $\rho_{\tau }=\frac{1}{3}$ and $\frac{1}{2}$. Similarly the position of the maximum in the δ=10 environment for $\rho_{\tau }=\frac{1}{2}$ is m=−0.2 in the extended framework model compared to m=−0.3 in the original model. However the curves are very flat around the peak, and the position of the maxima in both the extended and original frameworks can vary between neighbouring values of m from run to run, owing to stochasticity in the simulations, so the trends are more informative than the exact positions of the maxima. The overall pattern of behaviour remains qualitatively unchanged.

(Note that to simulate the evolution numerically, we need to calculate γ at each generation, for which we first need to calculate the phenotypic variance as in Appendix B of Hoyle and Ezard (2012). In doing so we neglect the update to $z_{t-2}^{*}$ at selection in generation t−1, which we expect to be a good approximation when ∣mγ∣ is small (i.e., for weak enough selection and/or ∣m∣ small enough), the same conditions under which the other approximations used in that same calculation hold. We further note a correction to the value of τ used in Fig. 2 of Hoyle and Ezard (2012), which was τ=0.20 not τ=0.25 as originally written. We have used τ=0.20 for our comparison here).

### Evolutionary Response to a Noisy Step Change

Next we investigate the response to a noisy step change environment $\varepsilon_{t}=\delta U_{t}+\xi_{t}$, where $U_{t}$ represents the value at time t of the unit step function that changes from 0 to 1 at time t=0, δ is the amplitude of the environmental step and $\xi_{t}$ is a Gaussian stationary autocorrelated random process as before. We take the mean over the distribution of environments, treating ${\bar{a}}_{t}$, ${\bar{b}}_{t}$ as fixed and neglecting correlations in the noise over periods of a generation or more, to obtain the evolution equations for the expected values of the genetic components and the phenotype after selection, which are

to $O\left(m\right)$, where $\gamma_{e}=1/\left(\omega^{2}+E\left(\sigma_{z_{t}}^{2}\right)\right)$ as in Hoyle and Ezard (2012), and following the approach there we approximate $E\left(\sigma_{z_{t}}^{2}\right)$ in the numerical simulations by the equilibrium expression in an environment $\delta U_{t-\tau }$. (Note that approximating these equations at $O\left(m\right)$ omits the $O\left(m^{2}\right)$ term that appeared in the bracket $\left({\bar{b}}_{t}-\rho_{\tau }B\right)$ in Hoyle and Ezard (2012). It is also equivalent to treating ${\bar{z}}_{t-1}^{*}$ as fixed when averaging over environments, and so to treating the history of environments up to and including the point of selection in the previous generation as fixed, while taking the mean only over environments after that point up to and including the point of selection in the current generation. This is arguably a more logical approach than in Hoyle and Ezard (2012), where we retained noise from $\varepsilon_{t-\tau -1}$ in ${\bar{z}}_{t-1}^{*}$. Since we are now treating ${\bar{z}}_{t-1}^{*}$ as fixed, we no longer write $E\left({\bar{z}}_{t-1}^{*}\right)$ in the equations as we did in Hoyle and Ezard (2012)). The approximations for $\gamma_{e}$ and the expected mean fitness in this Appendix and Appendix C assume $\sigma_{z}^{2},\sigma_{\xi }^{2}\ll \omega^{2}$.

The equation for $E\left(\Delta \bar{b}\right)=0$ in a noisy equilibrium environment $\varepsilon_{t}=\delta +\xi_{t}$ then gives

$$\bar{b}-\rho_{\tau }B=0,$$

and so the equilibrium states in the changed environment become

$$\begin{array}{c} E\left(\bar{a}\right)=\left(1-m\right)A+\left(1-m-\rho_{\tau }\right)\mathrm{\delta B},\\ E\left(\bar{b}\right)=\rho_{\tau }B, \end{array}$$

while

$$E\left(\bar{z}\right)=E\left({\bar{z}}^{*}\right)=A+\mathrm{\delta B}$$

is unchanged. The past states and current states have a consistent equilibrium to $O\left(m\right)$.

The expression for the expected mean fitness becomes:

where we have approximated to $O\left(m\right)$ and also neglected $O\left(m^{2}\gamma^{2}\right)$ terms in the second exponent arising from the presence of environmental terms in ${\bar{e}}_{t-1}^{*}$ within ${\bar{z}}_{t-1}^{*}$ (consistent with neglecting covariances between e and x that arise through selection).

We plot the expected evolution in response to an environmental step change in Figure B.2. As in Hoyle and Ezard (2012), we see that plasticity, $E\left({\bar{b}}_{t}\right)$, evolves to high values immediately following the step change and gradually dies away as the elevation, $E\left({\bar{a}}_{t}\right)$, adapts. Fitness drops following the step change but recovers quickly as the phenotype adapts; positive maternal effects speed up the process of adaptation, but lead to lower fitness in the long term.

Again the qualitative pattern of behaviour is very similar in the extended and original Hoyle and Ezard (2012) frameworks, and the fitness and phenotype trajectories are virtually unchanged. There are some small changes in the trajectory of the components $E\left({\bar{a}}_{t}\right)$ and $E\left({\bar{b}}_{t}\right)$ resulting from updating the evolution equations and approximating them at $O\left(m\right)$. The main impact comes from approximating the equilibrium state at $O\left(m\right)$, since the equilibrium value of $E\left(\bar{b}\right)$ is now the same for all values of m, whereas in our original model it was smaller for larger m. As a consequence the final equilibrium value of $E\left(\bar{a}\right)$ is reduced for larger m in the revised version so that the overall phenotype attains a value close to the optimum.

The remainder of the calculations in Hoyle and Ezard (2012)—the approximation to the expected mean fitness in eq. (3.4) and the stability calculations in Appendix C—can similarly be updated to reflect the extended theoretical framework, which would be expected to lead to small quantitative changes, but no significant qualitative impacts. Fig. 3 remains unchanged.

### Adaptation to Fluctuating Environments

In Ezard et al. (2014), we investigated the adaptation of phenotypes described by the Hoyle and Ezard (2012) model to fluctuating environments that change either fast or slowly, sinusoidally or stochastically.

Writing the environment in the form $\varepsilon_{t}=\Upsilon_{t}+\xi_{t}$, where $\Upsilon_{t}$ represents the long‐term (sinusoidal or stochastic) variation of the environment and $\xi_{t}$ is autocorrelated noise as before, the expected updates averaged over environments in the extended framework become

to $O\left(m\right)$, and the update for $E\left({\bar{z}}_{t-1}^{*}\right)$ becomes

To calculate $\gamma_{e}$ we now use the slightly different approximation to $E\left(\sigma_{\mathrm{zt}}^{2}\right)$ given in eq. (4) of Ezard et al. (2014).

Of particular interest are the components of the expected population mean fitness

$$E\left(\bar{W}\right)\approx W_{\max}$$

(B1)
$$\times \sqrt{\gamma_{e}\omega^{2}}$$

(B2)
$$\times \exp\left\{-\frac{\gamma_{e}}{2}{\left({\bar{a}}_{t}-A+\left(\Upsilon_{t-\tau }{\bar{b}}_{t}-\Upsilon_{t}B\right)+m{\bar{z}}_{t-1}^{*}\right)}^{2}\right\}$$

(B3)
$$\times \exp\left\{-\frac{\gamma_{e}\sigma_{\xi }^{2}}{2}\left({{\bar{b}}_{t}}^{2}+B^{2}-2{\bar{b}}_{t}B\rho_{\tau }\right)\right\},$$

where the components in Equation lines ((B1), (B2), (B3)) are the variance load, adaptation and fluctuation load respectively, and where again we have approximated to $O\left(m\right)$.

With the small changes to the evolution equations in the extended framework, we examine the contribution of each of these components to the expected fitness in slow (ν=0.01) and fast (ν=0.1) sinusoidal environments,

$$\Upsilon_{t}=5\sin\left(2\mathrm{\pi \nu t}\right),$$

and in environments that flip stochastically between $\Upsilon_{t}=-5$ and $\Upsilon_{t}=5$ as a fast or slow Poisson process (with times between flips exponentially distributed with mean μ=5 and μ=50 respectively). In all cases microenvironmental fluctuations with $\sigma_{\xi }=2$ were superimposed on top.

Results are shown in Figure B.3, which is the equivalent of Ezard et al. (2014) Fig. 3. Comparing this with a rerun of the original version shown in Figure B.4, we see that the behaviour is qualitatively and quantitatively very similar in the two cases. (Note that while Ezard et al. (2014) gives an $O\left(m\right)$ approximation to the evolution equations of Hoyle and Ezard (2012), the $O\left(m^{2}\right)$ terms were included in the simulations and we have done the same for the original model versions here.) The biggest differences can be seen in the adaptation and fluctuation load components of the fitness. The fluctuation load shows greater fitness costs for larger negative values of the maternal effect coefficient in the extended framework compared to the original version (compare Figures B.3d,h,l and B.4d,h,l), likely owing to there being no $O\left(m^{2}\right)$ terms in the plasticity update equation and fluctuation load component in the extended framework in contrast to the original model. Conversely the adaptation component shows less of a fitness cost for larger negative values of m, particularly in slowly changing environments (Figures B.3c, B.4c). These combine to give slightly higher fitness in the extended framework. However these effects are most evident at values of m close to the lefthand end of the range (m=−0.7) that are towards the more negative end of the range typically measured in reality. There are small shifts of some maxima in Figures B.3a,c,e,g,h,k, reflecting that the curves are very flat near the maxima rather than any real interpretive significance.

In keeping with the larger fitness costs of fluctuation load, it can be seen that the time averaged expected value of the slope of phenotypic plasticity, $E\left(\bar{b}\right)$, is larger at large negative m in the extended framework (Figure B.5) compared to the original version (Figure B.6). This greater within‐generation plasticity may explain the lesser fitness costs of adaptation for large negative m, which mean that in contrast to the original model adaptation is no longer the major influence on fitness in slowly changing environments and instead fluctuation load appears to be the most important factor (We do not plot the time averaged expected value of the elevation, since it is zero in the case of sinusoidal environments and depends on the particular realisation of the sequences of flips for stochastic environments).

## Appendix C Calculations for Fixed Maternal and Grandmaternal Effects

Here we repeat the analyses of Prizak et al. (2014) now using the extended framework.

Using Equations ((13), (14), (15)) and (16) the expectations of updates over the distribution of environments become to first order in m and g

where $\gamma_{\mathrm{em}}$ is the value of $\gamma_{e}$ in the maternal generation. Past phenotypes on the righthand side of the equations are effectively treated as fixed with respect to environmental noise, and we neglect correlations in the noise over periods of a generation or more. There is a consistent equilibrium in a constant environment for the expected present and past states to first order in m and g, such that

$$\begin{array}{c} E\left(\bar{a}\right)=\left(1-m-g\right)A+\left(1-m-g-\rho_{\tau }\right)\mathrm{\delta B},\\ E\left(\bar{b}\right)=\rho_{\tau }B,\\ E\left(\bar{z}\right)=E\left({\bar{z}}^{*}\right)=E\left({\bar{z}}^{**}\right)=A+\mathrm{\delta B}. \end{array}$$

In calculating $\gamma_{e}$ for use in the updates, we can use the expression for the variance at equilibrium given in eq. (8) of Prizak et al. (2014) even though expressions used in its derivation neglect updates to $z_{t-1}^{*}$ and $z_{t-2}^{**}$ at selection in the current generation, and to $z_{t-2}^{*}$ in the maternal generation, because these updates are zero at equilibrium and the final expression for the variance is not affected.

We approximate the expected fitness as follows:

to $O\left(m,g\right)$.

The evolution of the expected fitness in the extended framework shows the same qualitative pattern as in the original version with only minor quantitative differences (Figure C.1). The only noticeable change in the extended framework is that when m is negative, the return to equilibrium fitness is slightly faster.

(Note that we corrected bugs in the simulation code for the original version: reinstating the final exponential term, the fluctuation load term, in the expression for the expected mean fitness (eq. 9 of Prizak et al. (2014)) that had been missing and correcting the term ${\bar{b}}_{t}^{2}g^{2}$ there to ${\bar{b}}_{t-1}^{2}g^{2}$, and correcting a small error in the approximation to the expected phenotypic variance (eq. 8 of Prizak et al. (2014))).

The expected mean fitness at equilibrium and the time to recover equilibrium fitness (Figure C.2) show broadly the same qualitative picture with the fitness values in the extended framework being very similar to those in the original model framework, and the time to return to equilibrium fitness slightly faster over most of the domain. While m has a slightly greater impact than g on the time to recover fitness in the extended framework, as shown by the angle of the contour lines being slightly closer to vertical than horizontal, the same only appears to be true in the corrected original framework for some parts of the domain, such as the quadrant where both m and g are positive, but not others, for example in the bottom left corner where both m and g are towards the most negative end of their range and contour lines lie closer to the horizontal. Here we use an updated criterion for return to fitness, being the number of generations to return to and remain within 0.01 of the equilibrium fitness (defined as the fitness 100,000 generations after the step change). Using the original return to fitness criterion of returning to and remaining within 0.0001 of the equilibrium fitness the equivalents of Figures C.2B,D do look quite different and the contour patterns are more complex. The original criterion captures very long transients that are influenced by the form of the fluctuation load term in the fitness (that we had previously accidentally omitted), and since this is different in the two models, the long‐term return to fitness also looks different, particularly for negative m. However in practice these are fine details that are of minor significance to the overall dynamics. We decided to present a slightly relaxed criterion here for a more relevant comparison.

There is no change to the expected phenotypic variance in a constant environment (Fig. 4 of Prizak et al. (2014)) in the extended framework.

## Appendix D Derivations for the Case of Evolving Maternal Effects

### Derivation of the Evolution Equations for Evolving Maternal Effects

To second order in m, the average phenotype

$${\bar{z}}_{t}={\bar{a}}_{t}+{\bar{b}}_{t}\varepsilon_{t-\tau }+\bar{m_{t}z_{t-1}^{*}}+{\bar{e}}_{t}.$$

becomes

since a, b, m and e are assumed to be independent, and where we assume that $G_{\mathrm{mm}}∼O\left(m^{2}\right)$. The double asterisk in ${\bar{z}}_{t-2}^{**}$ indicates that we are considering the grandmaternal phenotype after selection in the maternal generation, which is given by

$${\bar{z}}_{t-2}^{**}={\bar{a}}_{t-2}^{*}+\Delta_{t-1}{\bar{a}}_{t-2}^{*}+\left({\bar{b}}_{t-2}^{*}+\Delta_{t-1}{\bar{b}}_{t-2}^{*}\right)\varepsilon_{t-\tau -2}+{\bar{e}}_{t-2}^{*}+\Delta_{t-1}{\bar{e}}_{t-2}^{*}+O\left(m\right),$$

where $\Delta_{t-1}$ denotes the update at selection in generation t−1. In the expression for ${\bar{z}}_{t}$ we have used that $\Delta_{t-1}{\bar{a}}_{t-2}^{*}$, $\Delta_{t-1}{\bar{b}}_{t-2}^{*}$ and $\Delta_{t-1}{\bar{e}}_{t-2}^{*}$ are independent of m to $O\left(1\right)$.

The small m approximation greatly simplifies the analysis compared to Kuijper and Hoyle (2015): the derivatives of ${\bar{z}}_{t}$ can be calculated directly as

The phenotype after selection in the current generation is given by

We use this to derive components of the phenotypic variance (Equation 18; eq. A6 of Kuijper and Hoyle (2015))

as follows

where we have first calculated

$$\bar{m_{t}z_{t-1}^{*}}={\bar{m}}_{t}\left({\bar{a}}_{t-1}^{*}+{\bar{b}}_{t-1}^{*}\varepsilon_{t-\tau -1}+{\bar{e}}_{t-1}^{*}\right)+\left(\frac{1}{2}G_{\mathrm{mm}}+{\bar{m}}_{t}{\bar{m}}_{t-1}^{*}\right)\left({\bar{a}}_{t-2}^{**}+{\bar{b}}_{t-2}^{**}\varepsilon_{t-\tau -2}+{\bar{e}}_{t-2}^{**}\right)+O\left(m^{3}\right),$$

and where we assume that $G_{\mathrm{mm}}∼O\left(m^{2}\right)$. Thus the phenotypic variance is approximated as

and to that approximation its derivatives are given by

Thus to $O\left(m/\omega^{2}\right)$ for ${\bar{a}}_{t}$ and ${\bar{b}}_{t}$ and to $O\left(m^{3}/\omega^{2}\right)$ for ${\bar{m}}_{t}$ the update equations become

where we have neglected terms of $O\left(m^{2},G_{\mathrm{mm}}\right)$ within the square brackets in the update equations, so that we are retaining terms at the two leading orders for each equation (except for the ${\bar{e}}_{t}$ update equation which is exact). Note that we also retain the implicit $O\left(m^{2}\right)$ terms in the products that involve $\left({\bar{z}}_{t}-\theta_{t}\right)$ so as to retain the functional structure of the equations.

Within the update equation for ${\bar{m}}_{t}$ we can approximate

Since we assume $m>>1/\omega^{2}$, and we know that

$${\bar{e}}_{t-1}^{*}={\bar{e}}_{t}+\Delta {\bar{e}}_{t}=\Delta {\bar{e}}_{t}∼O\left(1/\omega^{2}\right),$$

we can also neglect ${\bar{e}}_{t-1}^{*}$ in the update equation for ${\bar{m}}_{t}$ and in the expression for the phenotypic variance, and so to $O\left(m^{3}/\omega^{2}\right)$ we have

and to $O\left(m^{2}\right)$ we have

### Derivatives of the Heuristic Phenotypic Variance for Evolving Maternal Effects

The derivatives of the heuristic expression for the phenotypic variance (Equation 20) are given, to the approximation used there, by

Using these derivatives the update equation for ${\bar{m}}_{t}$ becomes

which upon substituting ${\bar{a}}_{t-1}^{*}={\bar{a}}_{t}$, ${\bar{b}}_{t-1}^{*}={\bar{b}}_{t}$ and ${\bar{m}}_{t-1}^{*}={\bar{m}}_{t}$ becomes
